# Effect of immunosuppression on hESC-derived retina organoids in vitro and in vivo

**DOI:** 10.1186/s13287-025-04271-z

**Published:** 2025-04-05

**Authors:** Robert Sims, Bin Lin, Yuntian Xue, Raghda Fouda, Bryce T. McLelland, Gabriel Nistor, Hans S. Keirstead, Andrew W. Browne, Magdalene J. Seiler

**Affiliations:** 1 Sue and Bill Gross Stem Cell Research Center, University of California Irvine, 845 Health Sciences Road, Irvine, CA 92697-1705 USA; 2 Ophthalmology, Gavin Herbert Eye Institute, University of California Irvine, Irvine, CA USA; 3 Physical Medicine and Rehabilitation, University of California Irvine, Irvine, CA USA; 4 Anatomy and Neurobiology, University of California Irvine, Irvine, CA USA; 5 Center for Translational Vision Research, University of California Irvine, Irvine, CA USA; 6 Biomedical Engineering, University of California Irvine, Irvine, CA USA; 7 Institute for Clinical and Translational Science, University of California Irvine, Irvine, CA USA; 8 AIVITA Biomedical, Irvine, CA USA

**Keywords:** Retinal transplantation, Retinal degeneration, Retinal organoids, Human embryonic stem cells, Immunosuppression, Tacrolimus, Mycophenolate Mofetil

## Abstract

**Background:**

Photoreceptor (PR) enriched retinal organoid (RO) sheets (human embryonic stem cell [hESC]-derived ROs) resulted in restoration of visual acuity in immunocompromised retinal degenerate (RD) animal models after transplantation. Further assessment of their clinical potential requires evaluation in immunocompetent RD disease models with effective immune suppression. We characterized safety and efficacy profiles of both donor tissues and prospective immunosuppressive treatments in vitro; and in vivo in immunocompetent RD rats (strain *SD-foxn1 Tg(S334ter)3Lav*).

**Methods:**

Retinal identity of ROs was validated by histology, flow cytometry and gene expression profiling, and their immunogenicity to sensitized human immune cells was measured by mixed lymphocyte reactions (MLR). We measured the effect of RO exposure for 1–4 weeks to therapeutic concentrations of our immunosuppressant drugs of choice on gene expression and metabolic function using quantitative PCR (qPCR) and functional and structural fluorescence lifetime imaging (FLIM), respectively. Immunocompetent RD graft recipients were immunosuppressed by implanted tacrolimus (TAC) pellets and mycophenolate mofetil (MMF) in food. In vivo, LCMS aided assessments of drug pharmacodynamics. Flow cytometry immunophenotyping and assay of post-surgery cytokines were used to assess and monitor drug efficacy. Retinal transplants were imaged in situ using optical coherence tomography (OCT) at defined time points post-surgery. Visual function was assessed by optokinetic tests (OKT) and superior colliculus electrophysiology recording. At study endpoints, immune cell infiltration and donor photoreceptor engraftment into host retinal architecture was evaluated by immunohistochemistry.

**Results:**

Immunosuppressive drugs have no negative effects on RO development and metabolism in vitro; and low alloreactivity of ROs determined by MLR may be predictive to that of human graft recipients. In vivo, minimum effective dosing ranges of TAC and MMF were determined. We characterized the mechanisms and critical immune populations implicated in rejection; and subsequently demonstrated their effective suppression in our xenograft RD model. OKT measured significant visual improvement after RO transplantation. Transplants developed most retinal cell types including photoreceptors; and integrated with the host retina. However, immunosuppression induced higher sensitivity to ketamine anesthesia.

**Conclusions:**

This study proves the concept that immunosuppression is likely tolerable in retinal transplantation and human stem cell therapy for retinal degeneration patients.

**Graphical abstract:**

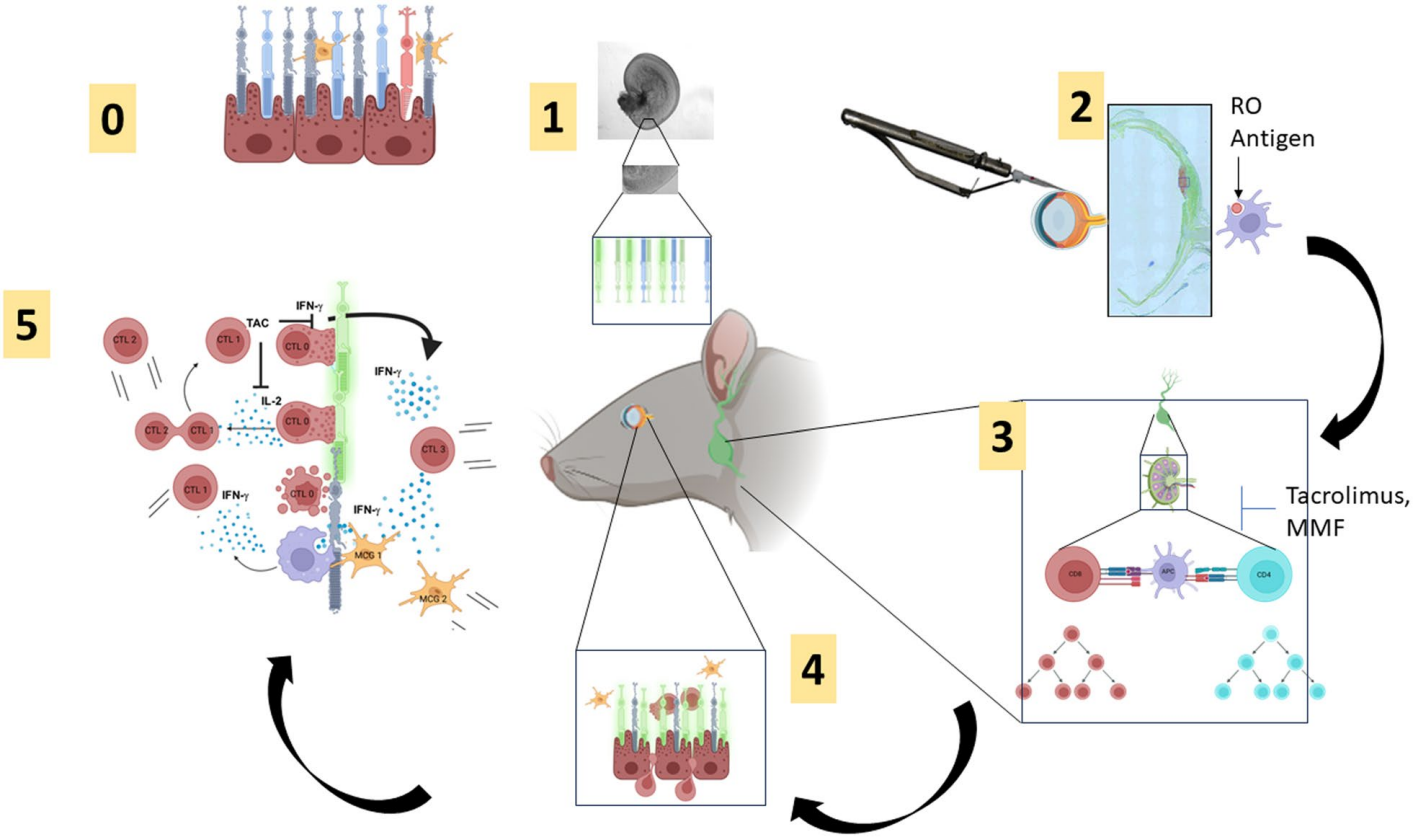

**Supplementary Information:**

The online version contains supplementary material available at 10.1186/s13287-025-04271-z.

## Background

Inherited retinal diseases (IRDs) of all causes have a prevalence of 1 in 3450 worldwide [1]. Specifically, RD caused by functional mutations such as Retinitis Pigmentosa (RP) which afflicts 1 in 4000 in the U.S. and 1 in 5000 worldwide [2, 3] have relatively early onset, quick progression to vision loss, and are more intractable to preventive measures and treatments. Restorative cell replacement therapies using stem cell derived photoreceptors have been explored as an investigative model and potential treatment option for replenishing diminished PR population and function resulting from RP, thereby serving as a possible renewable source of functional, non-mutant photoreceptors that may act to preserve visual response. Many investigators have developed protocols to differentiate pluripotent human embryonic stem cells (hESCs) into 3D retinal organoids (ROs). ROs develop the 3 principal types and strata of phototransduction neurons of the retina, photoreceptors, interneurons (bipolar, amacrine) and retinal ganglion cells [4–6]. Photoreceptors (rods and cones) are enriched in spherical 3D structures and comprise the laminated outer rim of the organoid.

Transplantation of PR enriched retinal organoid (RO) sheets has shown to improve visual function in several immunocompromised retinal degenerate animal models [4, 7, 8] (review: [9]) which produce no T-cells. “RN” rats with a rhodopsin mutation (*Rho S334ter-3*) and a *foxn1* mutation (strain *SD-foxn1 Tg(S334ter)3Lav*) provide a model to evaluate the regenerative capacity of the xenograft without risk of T-cell mediated immune response [10]. *Rho S334ter* line 3 rats, the RD rat model used in this study, express a mutation in a key terminal opsin amino acid (Serine residue 334) resulting in premature truncation of its assembly [11, 12]. This leads to loss of rhodopsin function in rods and their subsequent apoptosis by unfolded protein response mechanism [3]. Mass rod cell death leads to corresponding diminution of scotopic phototransduction and subsequently, functional impairment and death of cone photoreceptors. Rapid vision loss occurs as result of the domino effect of non-viable rods, and cone death resulting from a combination of ROS mediated oxidative stress, metabolic burden and cytotoxic byproducts of rod necrosis [13].

Non-nude (NN) RN rats undergo the same retinal degeneration driven by photoreceptor loss as nude RN rats except they have fully functional adaptive immune system with T-cells. Therefore, non-nude retinal degenerate rats receiving RO xenografts require immune suppression.

Research of clinical translational potential calls for evaluating the visual restorative capacity of RO transplants in both the context of RD *and* immune challenge, as the potential human beneficiaries of such retinal allografts will be immune competent and will—at least initially—require some degree of immunosuppressive therapy to prevent acute graft rejection. Thus, further study on the effect of immunosuppressants on retina organoids in vitro and in vivo (after transplantation) is necessary.

### Immunosuppressive drugs

In this study, we used tacrolimus and mycophenolate mofetil for immunosuppression. Tacrolimus (FK-506) is among a class of drugs called calcineurin inhibitors which downregulate transcription of IL-2, the cytokine essential for both the proliferation *and* activation of naïve T-cells. Tacrolimus diminished pan T-cell populations, most significantly T-helper and T-cytotoxic cells which are implicated in acute cell mediated xenograft rejection. Upon presentation with processed foreign antigen, naive T-cells undergo a signal transduction cascade culminating in the transcription of IL-2, whereupon the cytokine acts in both an autocrine and paracrine fashion to drive the activation *and* proliferation of both the secreting cell, as well as surrounding T-lymphocytes [14, 15].

By complexing with FK506-binding protein (FKBP) to inhibit calcineurin meditated activation of nuclear factor activated T-cells (NFAT), tacrolimus stops IL-2 production upstream in the signal transduction cascade, resulting in substantially impaired populations of functional antigen specific effector T-cells. As antigen specific antibody production depends on B-cell education by functional follicular T-helper cells, tacrolimus attenuates the humoral antibody mediated arm of adaptive immunity as well [16].

Mycophenolate mofetil (MMF) is a guanosine inosine monophosphate dehydrogenase inhibitor, which reversibly and non-competitively inhibits incorporation of purine nucleotides in a pathway largely utilized by B and T lymphocytes for mitosis. Thus, MMF is a drug that is cytostatic to B and T lymphocytes [17, 18]. MMF is hydrolyzed into its metabolite mycophenolic acid (MPA), used for in vitro drug testing.

### The role of microglia in Retinitis Pigmentosa

Microglia cells play distinct and integral roles in the retina. In healthy retina, microglia are primarily active in synaptic remodeling/pruning, the phagocytosis of apoptotic cells, immune surveillance, and are sparsely distributed in the inner nuclear layer (INL). Conversely, as Makabe and colleagues [19–22] observed, the natural history of microglia in RP is characterized by a dynamic staging in retina colocalization and activation status throughout the disease course, where microglia proliferate and localize to the outer retina and outer nuclear layer (ONL) to phagocytose cell bodies of mutant non-viable rod photoreceptors undergoing apoptosis. The subsequent complement mediated microglial activation and phagocytotic activity leads to a feed forward cascade of pro inflammatory cytokines and reactive oxygen species (ROS) that resulting in a neurotoxic, oxidative stress laden environment that further negatively impacts the survival and function of cone photoreceptors and RPE alike [23, 24]. Thus, this para-inflammatory condition in RP is the overarching context within which any subsequent ocular immune reactions in RP treatment and intervention must be evaluated.

### Immunodeficient *foxn-1* mutant

The transcription factor* foxn-1* is essential for thymic development, education, and maturation of functional T-cells. Its mutation leads to immunodeficiency characterized by thymic atrophy with absence of T-cells. This renders the adaptive component of antibody-mediated humoral immunity nonfunctional, as B-cell maturation and learned development of pathogen specific antibodies is contingent upon T-cell help [25]. Because female nude rats do not lactate, non-nude (*foxn1*+*/−*) females are used for breeding. Therefore, about half the progeny of our *Rho S334ter*, *foxn-1* mutant crosses are immune compromised, athymic (do not develop mature T-cells) and nude while the remainder are immune competent.

For our RO transplant study, the immune competent RD progeny provide a parallel age-matched comparison cohort to their immune compromised siblings—ideal to characterize the immunogenicity of RO xenografts and to model an effective immunosuppression regimen.

### Determining immunosuppressive drug effects by immunophenotyping

Immunophenotyping (differential census of lymphocyte populations) using flow cytometry served as a quantitative means of monitoring efficacy of immunosuppressant drugs (Tacrolimus (TAC), Mycophenolate Mofetil (MMF)) administered at (1) a specific dose and (2) multiple time points throughout the dosing period.

We define drug efficacy broadly as the degree of suppression of lymphocyte populations in treated relative to non-treatment controls. Furthermore, the efficacy of drug treatment is more narrowly defined by the prevention of acute rejection and promotion of long term transplant survival.

In vitro, we examined the effect of clinically determined therapeutic serum levels of TAC and MMF individually and in combination, upon RO differentiation, proliferation, and metabolic activity. In vivo, we monitor and characterize the immune phenomenon of subretinal xenograft rejection after subretinal transplantation of hESC-derived RO xenografts into retinal degenerate *Rho S334ter-3* rats. We then delineated insufficient from successful immune suppression using our drugs of choice, TAC, and MMF.

In vivo, we expect a larger immune challenge to hESC-derived tissues in an immunocompetent RD animal model as a xenograft than in prospective human subjects as an allograft. The allograft situation was modeled in vitro using mixed lymphocyte reactions with human PBMCs.

This study is translational research aimed at restoring vision in blind patients. Our previous work showed that transplantation of hESC derived retinal organoid (RO) sheets improved visual function in several immunocompromised retinal degenerate animal models [4, 7, 8] (review: [9]). Thus, this study focused on testing the effect of immunosuppressants on retina organoids in vitro and in vivo in an immunocompetent RD animal model.

## Material and methods

The work has been reported in line with the ARRIVE guidelines 2.0.

### Cell culture/RO development

CSC14 hESCs (supplier: AIVITA Biomedical, Irvine, CA) were expanded in custom formulated xeno-free medium (ABstem, FUJIFILM Irvine Scientific) supplemented with bFGF and Activin A (supplier Peprotech, now Thermo Fisher Scientific), in TC flasks coated in Matrigel (Corning). Upon reaching 70–80% confluency, colonies were dissociated with collagenase IV (2 mg/ml) and were subsequently passaged. To initiate differentiation, cells were allowed to aggregate into 3D embryoid bodies (EB) in suspension culture. Neural differentiation was initiated by transitioning to basal ABstem media containing Vitamin A-free B27 supplement (Gibco) without growth factors (Retinal differentiation media, RDM). On day 7 of differentiation, EBs were then plated on Matrigel (1:100; Corning; Thermo Fisher Scientific). At a density of 20–100 aggregates/cm^2^, fed 10 mL/ dish of RDM every other day. And maintained for 21–36 days. Upon appearance of laminated eye field structures, visible using low power brightfield and phase contrast microscopy, eye field structures were manually excised from Matrigel and placed in suspension culture. From day 55 of culture and for duration of in vitro maintenance, ABstem media containing B27(Gibco) supplement with vitamin A and 10% v/v fetal bovine serum was used (details of procedure described in [4]) (for reagents, see Supplemental Table S1). ROs were fed every 2–3 days as needed.

From each lot, sets of ROs were selected for potential in vivo study/photoreceptor tissue transplantation. Selected ROs were dissociated using a papain dissociation kit (Worthington Biochemical) and following manufacturers’ instructions. Viability of dissociated ROs was determined using trypan blue exclusion. From here, the ROs that were prospective tissue transplant source subsequently underwent further selection, based on the presence of a laminated photoreceptor layer as determined in brightfield microscopy.

### Mixed lymphocyte reactions

Retina organoids (ROs, n = 9) of three different lots were tested for in vitro immunogenicity using a two-way mixed lymphocyte reaction (MLR) [26]. The ages of the ROs were day 60, day 54, and day 49 of differentiation (similar to the tissues being transplanted in vivo). Human blood cells were GMP grade and fresh, not frozen; normal leukopacks (not mobilized) obtained from a commercial source (HemaCare, now Charles River Labs) and collected from healthy consented donors by leukapheresis. PBMCs were then isolated from whole blood using a Percoll density gradient. PBMCs are a mix of various immune cells including professional APCs and T cells. ROs were mixed with APCs (macrophages, dendritic cells, and monocytes) and T-cell responders for 7 days in standard cell culture conditions (37 °C, 5% CO_2_, 3 × media changes/week) (see Supplemental Table S1 for media). Control conditions included irradiated human dermal fibroblasts (supplier ATCC) and anti-CD3 microbeads (Dynabeads, Thermofisher). After 7 days, cells were collected and stained using Zombie Dye Violet Blue for live/dead cell discrimination post-fixation in 2% paraformaldehyde + 0.1% Saponin (Thermo-Fisher Sci). At the completion of the staining, cells were then incubated with fluorochrome-conjugated primary antibodies (human CD45, CD3, CD4, CD8, MHC-I, MHC-II, and Ki67) (Supplemental Table S2), and analyzed on a Novocyte flow cytometer (Acea Biosciences) for protein expression levels.

### Histology of ROs

Select ROs from each batch were fixed with 4% Paraformaldehyde, paraffin-embedded, and cut into 10 µM sections. Prior to antibody staining, sections were immersed in Xylene baths with gradual rehydration using decreasing concentrations of ethanol and finally rinsing with water. The rehydrated sections were then stained with anti-RAX (Abcam) and developed using DAB HRP kit (Vector Labs). The sections were then imaged, and the number of positive nuclei was then quantified manually. Multiple sections from several batches were characterized.

### Organoid drug study by fluorescent lifetime microscopy (FLIM)

To test the influence of immunosuppressant drugs on ROs’ metabolic activity, two batches of organoids at different stages of differentiation (starting days: day 68 and day 87) were used for FLIM imaging at two different timepoints (treatment duration for 1 week and 4 weeks). Three conditions were prepared by adding drug into the organoid culture medium for the duration of the experiment: (1) 0.5 ug/mL MPA, (2) 3 ng/mL TAC, and (3) combination of 0.5 ug/mL MPA and 3 ng/mL TAC. ROs were suspension cultured in ultra-low attachment 24-well plates (Corning Costar) (Corning, Corning, NY, USA), one RO per well with 2 mL culture medium. ROs were fed three times a week, each time replaced half of the culture media.

FLIM imaging method was the same as described in previous publications [5, 27]. Briefly, we used a Zeiss LSM 780 microscope with a Plan-Apochromat 20x/0.8 M27 objective (Carl Zeiss, Jena, Germany) to acquire images on the live ROs’ cross-section. The laser excitation wavelength was 740 nm. The metabolic activity was quantified using the free and bound intrinsic fluorophore nicotinamide adenine dinucleotide (NADH) ratio, which is a metric derived from the phasor approach developed by Digman et al. [28]. Free NADH was linked to more glycolysis and a more proliferative state, while bound NADH was correlated with more oxidative phosphorylation and a more differentiated state [29]. Each treatment group contained 4 ROs for measurement and one-way ANOVA analysis was performed.

### Quantitative polymerase chain reaction (qPCR) analysis

**Fig. 1:**
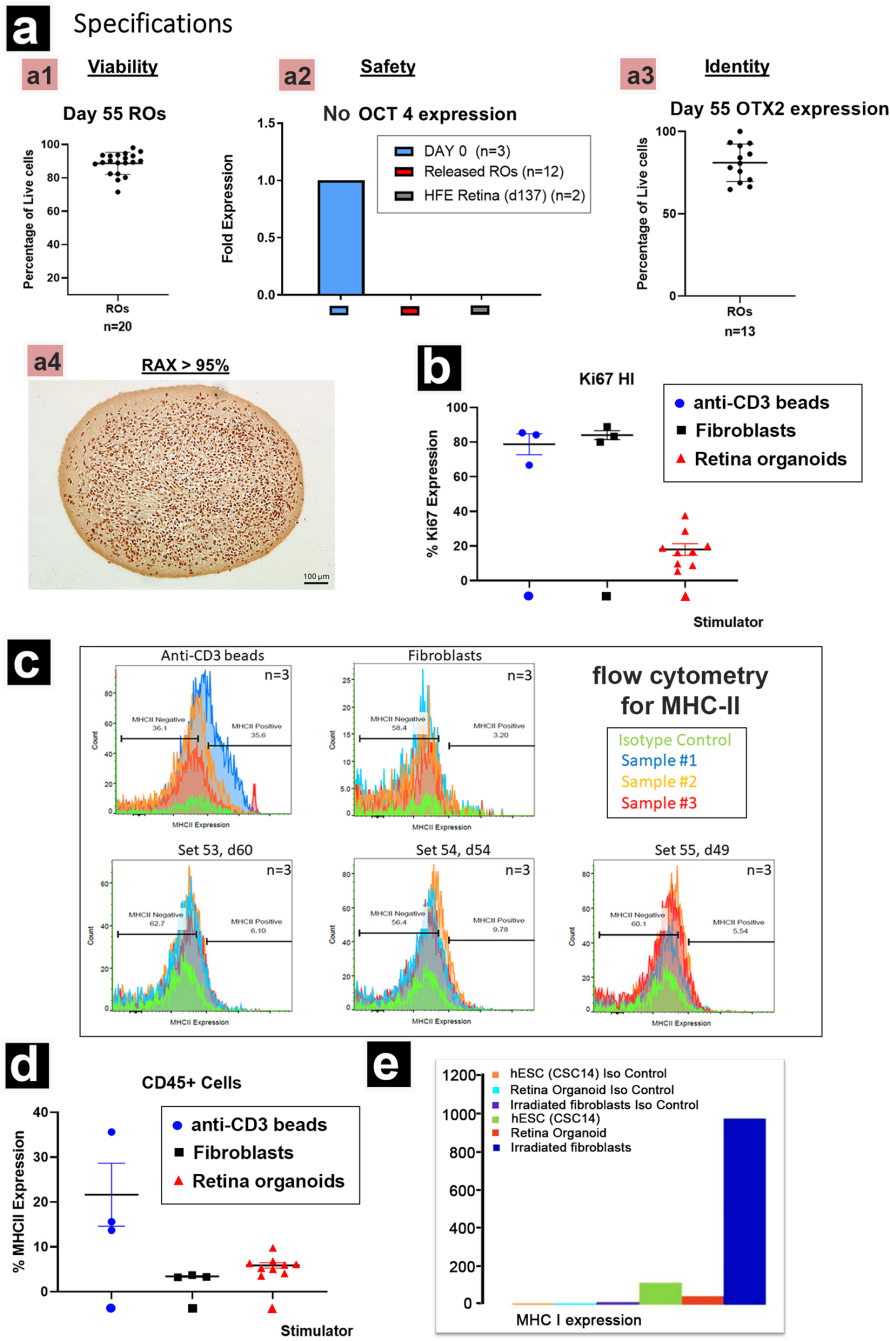
Retinal organoid (RO) specifications and mixed lymphocyte reactions: **a** Specifications of lot release: **a1** lot viability (trypan blue test); **a2** No OCT4 expression in organoids (qPCR). Stem cells (d0, n = 3) compared with released ROs (n = 12, age 49–60 d) and human fetal retina (HFE) at d137 (n = 2); **a3** RO cells express > 75% OTX2 (flow cytometry); **a4** paraffin section of RO stained with anti-Retina and Anterior Neural Fold Homeobox (RAX) antibody. RAX is a transcription factor essential to retinal development and its expression is ubiquitous in ROs. Every nucleus in a differentiated organoid expresses RAX. **b** Mixed lymphocyte reactions. Retina organoids (ROs, n = 9) of three different lots were evaluated for in vitro immunogenicity using a two-way mixed lymphocyte reaction (MLR). After 7 days of co-culture, live T-cells (CD45+ CD3 +) were stained with Ki67. Protein expression was analyzed using flow cytometry. High expression of the proliferation marker Ki67 is indicative of a T-cell response. Control conditions included irradiated human fibroblasts (n = 3) and anti-CD3 microbeads (n = 3), both of which induced a strong response. The retina organoids (n = 9) did produce some proliferation, but it was significantly reduced as compared to controls (*p* < 0.0001). **c** Mixed lymphocyte reactions. Cells were gated as live and CD45 positive. The MHC-II expression levels of the responders were measured after 7 days of co-culture. The retina organoids did produce some proliferation, but significantly less compared to αCD3 microbead controls (*p* < 0.0012). **d** Graph of flow data shown in (**c**), showing the low response of lymphocytes to ROs. **e** Significantly reduced expression of MHC-I in retina organoids in comparison to hESC and irradiated fibroblasts

**Fig. 2:**
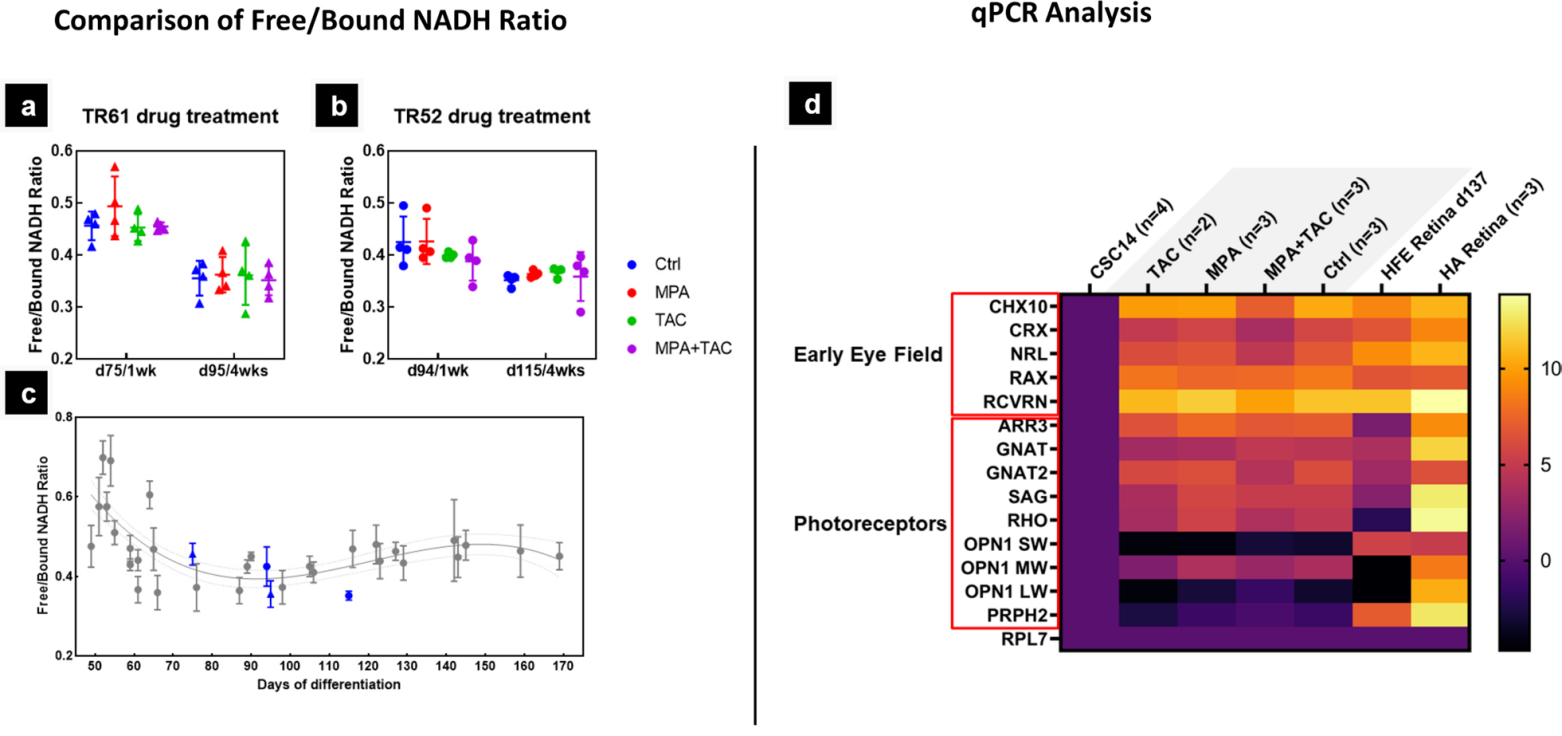
Influence of immunosuppressant drug on the long-term metabolic activity of retinal organoids. 2P microscopy (fluorescence lifetime [FLIM] analysis) was used to show shift from glycolytic to OXPHOS based metabolism, measured as ratio of free to bound NADH. **a** and **b** Two different sets of organoids were evaluated after 1-week and 4-week exposure to MPA (red, 0.5 μg/ml), TAC (green, 3 ng/ml) or a combination of both (purple) at the lowest effective concentration reported for human clinical trials. No significant difference in free/bound NADH was detected (one-way ANOVA) between drug-treated organoids and controls. **c** Overview of average free/bound NADH profiles among RO lots across days 50–170 of differentiation. Grey data points represent a modified format from Fig. 1b of previous publication [5], control groups for drug treatment are colored in blue. Control samples appear to correspond to the previously reported samples in [5]. **d** mRNA expression heatmap across IS drug exposed ROs. TAC and MPA treated samples showed similar gene expression level as control. The combined treated sample showed a discernable fold change in downregulation of CRX and NRL compared to the other three groups. Retina organoids’ age ranged from 13.5 to 16.5 weeks (day 95–115). Drug-treated for 4 wks. log2 F.E − log2(fold expression). Ctrl = control; MPA = Mycophenolic acid (0.5 µg/ml); TAC = Tacrolimus (3 ng/ml)

The primers for qPCR test are listed in Supplemental Table S3 (Qiagen, Germantown, MD, USA). In total, 14 retinal progenitor and photoreceptor genes and one housekeeping gene used as reference gene were examined for gene expression profiles. Human fetal (HFE, age 137 days = 4.6 months) and adult retinal (HA) tissue (Eye bank, UCI-20–153-C-T) were used as positive controls. For organoid specification (Fig. 1a2), 12 RO samples between day 49 and 60 of differentiation were used. For drug treatment analysis (Fig. 2d), ROs were used after 4 weeks of drug or control treatment (differentiation age day 95–115). Each sample set consisted of 3–5 ROs.

Trizol reagent (Qiagen), DNase I digestion (Invitrogen, TURBO, Waltham, MA, USA), and phenol–chloroform extraction (Fisher) were used to isolate RNA. A RT2 cDNA synthesis kit (Qiagen) was used to synthesize cDNA. RT2 SYBR Green with ROX qPCR master mix (Qiagen) was used for amplification, which was performed with the following protocol: 95 °C (15 min), 40 cycles at 95 °C (15 s each), 55 °C (30 s each), and 72 °C (30 s each). The annealing temperature was 60 °C. The double delta cycle threshold (Ct) method was used to calculate the fold-expression with day 0 undifferentiated hESC (line CSC14) as the negative control. For analysis and heatmap generation, non-detected amplification in the control tissue and ROs were assigned cycle threshold values of 40. Heat maps with values in log_2_ (Fold Expression) were generated using Graphpad Prism software (Graphpad Software LLC, La Jolla, CA, USA).

### Animals

Animals were treated in accordance with the National Institutes of Health guidelines for the care and use of laboratory animals and the ARVO Statement for the Use of Animals in Ophthalmic and Vision Research, and under a protocol approved by the Institutional Animal Care and Use Committee of the University of California-Irvine (AUP18-145). All animals were group-housed (two or three rats per cage) unless veterinary care necessitated individual housing, in cage racks with individually filtered air, and provided with EnviroDri and rat tunnels. All rats were maintained on a 12-h light/dark cycle (lights on from 6:30 am to 6:30 pm) at an ambient temperature of 21.5 ± 0.88 °C and relative humidity of 50%. As previously reported, donor retinal transplant tissue was obtained from retinal organoids expanded from CSC14 hESCs. *SD-foxn1 Tg(S334ter)3Lav* (RD nude rat) transplant recipients were previously generated by crossing *SD-Tg(S334ter)3Lav* rat and *NTac:NIH-Whn* rats [10]. Therefore, recipient rats have a mutant rhodopsin gene and a T-cell deficiency resulting in immunocompromised and retinal degenerate rats. Non-nude *(foxn1*+*/−*) females were mated with nude (*foxn1−/−* males, resulting in litters with 50% nude and non-nude pups. A total number of 20 Long-Evans rats, 151 non-nude immunosuppressed (IS) RD rats; and 18 non-nude non-IS RD rats of either sex were used for this study (see Tables 1, 2, 3). Thirteen immunodeficient RD rats were selected from a larger study involving 177 immunodeficient RD rats (see Table 4).

**Table 1:** Overview of immunosuppression dosing experiments

IS dosing (LE and “RN” rats) = 35 rats (18M, 17F)			
Rat type	Treatment	Duration	Outcome
4 LE rats (2F, 2M) 1 “RN” rat (M)	None (control)	n/a	Blood collection up to age 80–84 d
4 LE rats (2F, 2M)	5 mg 90-d release TAC @ age 23d	57 d	Blood collection up to age 80 d
4 LE rats (2F, 2M) 4 “RN” rats (2F, 2M)	10 mg 90-day release TAC @ age 22d	15–57 d	Blood collection up to age 79d (1 LE rat died @ age 37d 1 “RN” rat died at 41d)
8 LE rats (4F, 4M)	10 mg 90-day release TAC + MMF 100 mg/kg in food, starting @ age 22d	57 d	Blood collection up to age 79d 2 rats (1M, 1F) died at age 48 and 71d
3 “RN” rats (1F, 2M)	MMF dosing (200 mg/kg food) starting @ age 65d	62–83 d	Blood collection up to age of 133–148 d
3 “RN” rats (2F, 1M)	MMF dosing (150 mg/kg food) starting @ age 65d	68–77 d	Blood collection up to age of 133–142 d
4 RN rats (2F, 2M)	20 mg 90-day release TAC + MMF 300 mg/kg, then 200 mg/kg in food, starting @ age 39d	70–77 d	Blood collection up to age of 109–116 d

**Table 2:** Overview of immunosuppression pilot experiments

Rat type	Treatment	Age at surgery	Days post-surgery (dps)	Age at death	Died on surgery day	Died pre-maturely	Cornea issues	Surgical trauma	Outcome
Non-immunosuppressed rats (NN NO IS) = 18 rats (16M, 2 F)—pilot experiments									
Transplant (1F, 11M)	None	37–42 d 38.1 ± 0.7)	0–77 d (70.9 ± 8.5)	36–114 d (70.9 ± 8.3)	2/12 (17%)	2/12 (17%)	1/12 (8.5%)	2/12 (17%)	TP rejection (43–77 dps) blood collection; histology
Sham (4M, 1F)		37 d (37.0 ± 0)	51–188 d (142.8 ± 25)	88–225 d (179.8 ± 25)	0	0	2/5 (40%)	0	blood collection; histology
AMC (1M)		n.a	n.a	104 d	n.a	n.a	n.a	n.a	blood collection; histology
IS rats TAC only (NN IS TAC) = 15 rats (10F, 5 M)—Pilot experiments									
Transplant (8F, 1M)	10–20 mg 90-d TAC	35–38 d (35.8 ± 0.3)	0–82 d (33.4 d ± 9.8)	36–117 d (69.2 d ± 9.8)	2/9 (22%)	4/9 (44%)	2/9 (22%)	1/9 (11%)	TP rejection: 2/9 (22%)
Sham (2M)		35–37 d (36 d ± 1.0)	0–69 d (34.5 ± 34.5)	36–104 d (70.0 ± 34.0)	1/2 (50%)	1/2 (50%)	0	0	Blood collection, histology
IS rats MMF only = 12 rats (6M, 6F)—Pilot experiments									
Transplant (4F, 5M)	100–300 mg/kg MMF in food	35–44 d (39.7 ± 2.9)	7–41 d (27.7 ± 2.9)	51–85 d (67.6 ± 3.1)	0	0	2/9 (22%)	2/9 (22%)	TP shrinking in OCT; TP rejection 1/9 (11%);
Sham (2F)		44 d (av. 44)	41 d (av. 41)	74–85 d (79.5 ± 5.5)	0	0	0	0	Blood collection; histology
AMC (2M)		n.a	n.a	66–233 d (149.5 ± 83.5)	n.a	0	0	n.a	Blood collection, histology

**Table 3:** Overview of main immunosuppression experiments

Rat type	Treatment	Age at Surgery	Days post-surgery (dps)	Age at death	Died on surgery day	Died pre-maturely	Cornea issues	Surgical trauma	Outcome
IS rats TAC + MMF = 77rats (42M, 35F)—main experiments									
Transplant (20F. 29M) *donor age* 39–99 d (62.2 ± 2.4)	20–30 mg TAC (repeated every 60–90 d); 300 mg/kg MMF in food	30–50 d (38.1 ± 0.7)	0–190 d (50.3 d ± 9.0)	33–224 d (88.4 ± 9.1)	11/49 (22.4%)	35/49 (71.4%)	7/49 (14.2%)	4/49 (8.2%)	SC recording 2 rats; 3 rats died before they could be recorded Blood collection; visual testing; histology
Transplant long survival (> 100 d age) (8F, 8M) *donor age* 39–99 d (64.1 ± 4.0)		30–43 d (37.6 ± 1.5)	70–190 d (130.8 ± 10.9)	102–224 d (168.4 ± 11.4)		9/16 (56.25%)	4/16 (25%)	2/16 (12.5%)	
Sham (11F, 9M)		36–43 d (39.3 ± 0.7)	0–182 d (64.0 ± 15.9)	37–232 d (103.5 ± 16)	3/20 (15%)	9/20 (45%)	2/20 (10%)	3/20 (15%)	SC recording 3 rats; 1 rat died before it could be recorded Blood collection; visual testing; histology
Sham long survival (> 100 d age) (4F, 3M)		38–42 d (40.6 ± 0.6)	71–211 d (142.0 ± 14.1)	109–225 d (182.9 ± 13.9)		1/7 (14.3%)			
AMC (4F, 4M)		n.a	n.a	67–289 d (128.5 ± 34.2)	n.a	3/8 (37.5%)	n.a	n.a	SC recording 3 rats; 1 rat died at TAC re-implant
AMC long survival (> 100 d age) (3F, 3M)		n.a	n.a	121–289 d (195.2 ± 34.0)	n.a	1/6 (16.7%)	n.a	n.a

**Table 4:** Selected immunodeficient RD rats (used for blood draws and histology comparisons) (details in another paper)

Rat type	Age at Surgery	Days post-surgery (dps)	Age at death	Analysis
Transplant (10F, 2M) donor age 37–79 d (58.5 ± 4.7)	35–44 d (37.6 ± 0.8)	40–377 d (205.3 ± 15.5)	78–377 d (242.9 ± 15.5)	Blood collection in 2 rats (40 and 215 dps) SC recording in 10 rats SC responses in 6 rats istology, immunohistochemistry
AMC (2M)	n.a	n.a	235 d	Blood collection histology

### Immunosuppressant drug administration

Tacrolimus was administered in the form of pellets intended for subcutaneous implantation and formulated for controlled quantity of drug release per unit time specified (e.g., 20 mg/ 90 day release). Original supplier for Tacrolimus was EuroAsias Group Inc. (Edison NJ, U.S.A., catalogue number EGI-052). Innovative Research of America (Sarasota FL USA) then made time-release pellets. As such, each dose and time period release specified, guaranteed a fixed quantity of drug released for daily absorption, circumventing potential fluctuations in daily dosing variations introduced by more labor-intensive methods like oral gavage. This method also provided a constant variable necessary to evaluate the drugs kinetic profile.

Immunocompetent Long-Evans (n = 16) or RD rats designated for immunosuppression (n = 115; 70 transplants, 36 sham surgeries, 45 non-surgery rats) received a subcutaneous implant of tacrolimus pellet, as described in [30], ranging from 20 to 40 mg and from 60 to 90-day slow-release delivery (Innovative Research of America, Sarasota, FL USA). This resulted in theoretical dosing regimen ranges from 0.74 to 7.14 mg/kg/day, which at its highest parameter exceeds the recommended dose for rat 0.1–1 mg/kg/day [31], but is well below LD50 of 23.6 mg/kg. An overview of dosages is provided in Table 5.

**Table 5:** TAC and MMF dosing experiments

*TAC dosing*					
Weight range 70–300 g*					
0 Tx	10 mg 90-day TAC pellet	20 mg 90-day TAC pellet	20 mg 60-day TAC pellet	30 mg 90-day TAC pellet	30 mg 60-day TAC pellet
	0.37–1.59 mg/kg/day	0.74–3.17 mg/kg/day	1.11–4.76 mg/kg/day	1.11–4.76 mg/kg/day	1.67–7.14 mg/kg/day

*MMF dosing*					
Rat weight (g)	Consumption rate %BW	Consumption/day in (g)	100 mg/kg	150 mg/kg	300 mg/kg
100	8	8	8 g food @8 g(0.1)kg = 0.08 kg food × (100 mg) = 8 mg/kg dose (0.1 kg) = 0.8 mg/kg /day	8 g food @ (0.1)kg = 0.08 kg food × (150 mg) = 12 mg/kg dose (0.1 kg) = 1.2 mg/kg/day	8 g food @(0.1)kg = 0.08 kg food × (300 mg) = 24 mg/kg dose (0.1 kg) = 3 mg/kg/day
200	6	12	= 2.4 mg/kg/day	= 3.6 mg/kg/day	= 7.2 mg/kg /day
300	5	15	= 4.5 mg/kg /day	= 6.75 mg/kg/day	= 13.5 mg/kg /day

*Dosing regimens*								
	0	1	2	3	4	5	6	7
IS Tx	0 Tx	10 mg 90-day TAC	100 mg/kg MMF	10 mg 90-day TAC 150 mg/kg MMF	20 mg 90-day TAC	20 mg day TAC 150 mg/kg MMF	20 mg 60-day TAC 300 mg/kg MMF	30 mg 60-day TAC 300 mg/kg MMF
Dose daily in mg/kg	0	0.37–1.59 mg/kg/day	0.8–2.4 mg/kg/day	TAC 0.37–1.59 mg/kg/day MMF 1.2–6.75 mg/kg/day	TAC 0.74–3.17 mg/kg/day	TAC 0.74–3.17 mg/kg/day MMF 1.2–6.75 mg/kg/day	TAC 1.11–4.76 mg/kg/day MMF 3–13.5 mg/kg/day	TAC 1.67–7.14 mg/kg/day MMF 3–13.5 mg/kg/day

*Based on average initial weight range of NN TP IS at study entry (pellet implant) and endpoint

1 g of Mycophenolate mofetil (MMF) was obtained from LGM Pharma (Erlanger, KY, USA) or Focus Biomolecules (Plymouth Meeting, PA, USA) and fortified into custom formulated rat chow (Bioserv, San Diego, CA, USA) from 100 to 300 mg/kg. Chow was administered to each surgery cohort 1 week before transplantation and continuously thereafter. Formulation for average consumption across relevant ranges of body weight yielded dosing ranges from 12 to 13.5 mg/kg/daily, whereas Diehl et al. reported the recommended effective MMF dosing for rats was up to 30 mg/kg/day [31].

Each animal undergoing subcutaneous tacrolimus pellet implantation was anesthetized with a 2–3% isoflurane-oxygen mixture. An oval shaped area approximately 3 cm in width and 4 cm length was shaved behind the neck and surgical site antisepsis was performed. While under anesthesia, rats were transferred to a sterile surgery site and onto a heating pad, where a subcutaneous compartment of appropriate depth to accept pellet was made with an approximately 1 cm incision followed by extensive pruning of associated connective tissue into which tacrolimus pellet was inserted. The implant site was sutured with bio-absorbable sutures (VICRYL, Ethicon, Raritan, NJ, USA) and tissue glue (GLUture, Zoetis Inc., Parsippany, NJ, USA) was applied along the seams of the initial incision. Rats were administered buprenorphine 0.01 mg/kg (Reckitt Benckiser Pharmaceuticals, Inc., Richmond, VA, USA; Patterson Veterinary, Loveland CO, USA) post-procedure and prior to recovery. Topical triple antibiotic ointment was applied initially post-surgery and as needed thereafter.

### Blood collection

Blood samples for LCMS and flow immunophenotyping were collected by tail vein puncture. Rats were weighed to establish maximum blood collection volume (1%/body weight) and were restrained using Decapicone rodent restraint device (Braintree Scientific, Braintree, MA, USA), with tails left exposed. Chemical hand warmers were applied to encourage blood flow. Warmed tails were then suspended off edge of surface during venipuncture to promote optimal gravity assisted flow of blood into collection tubes. Tail veins were punctured with 25-gauge needle (BD) at a shallow angle where sample flow into needle hub was collected by capillary action into mini collection tubes with EDTA anticoagulant (Sarstedt, Newton, NC, USA) and either immediately processed for downstream procedures (centrifuge aided plasma separation for ELISA, lymphocyte isolation for immune phenotyping) or stored at − 80 °C for LCMS.

### Transplantation surgery

Transplant recipients were 145 RD rats (either sex) on postnatal day 30–50 (9 immunodeficient, 70 IS, 12 non-IS) which were randomly assigned to surgery (transplant or sham) and non-surgery groups within litter mates. Sixty-one rats were sham surgery controls (35 immunodeficient, 17 IS, 7 non-IS). Fifty-four rats were used as non-surgery controls (AMC) (2 immunodeficient, 36 IS) (see Tables 1, 2, 3, 4). Rats received 4 mg/kg ketoprofen (Zoetis, Inc., Parsippany, NJ, USA) and were subsequently anesthetized with ketamine xylazine (KX) solution (40–55 mg/kg Ketamine, 6–7.5 mg/kg Xylazine, sometimes with additional 2–3 mg/kg Acepromazine (KXA)). Pupils of both eyes were dilated with 1% atropine solution (Akorn pharmaceuticals), followed by dexamethasone drops (Bausch & Lomb, Inc., Tampa, FL, USA) for topical anti inflammation, and ophthalmic betadine solution (Alcon, Fort Worth, TX, USA) for surgery site antisepsis.

Each donor retinal tissue transplant was carefully dissected (under sterile conditions in culture media) from a selected RO with suitable photoreceptor lamination determined by bright field microscopy. The resulting donor tissue sheet (up to 1.3 mm × 0.8 mm) was loaded into a custom subretinal implantation instrument [4] and was delivered to the subretinal space of the left eye. For recovery, rats were given a subcutaneous injection of Ringer’s saline solution and buprenorphine (0.03 mg/kg) (Reckitt Benckiser Pharmaceuticals, Inc., Richmond, VA, USA; Patterson Veterinary, Loveland CO, USA) for pain management and placed in a Thermocare (Paso Robles, CA, USA) incubator to recover.

### Spectral domain optical coherence tomography (SD-OCT) imaging

Eyes of rats were imaged by SD-OCT using the Bioptigen Envisu R2200 Spectral Domain Ophthalmic Imaging System (Bioptigen, Research Triangle Park, NC, USA), initially at 14 days post-surgery and subsequently every 1–2 months thereafter. Designated animals were anesthetized by intraperitoneal injection with either ketamine/xylazine saline solution (KX) at 37.5 mg/kg ketamine and 5 mg/kg xylazine, or 40 mg/ml ketamine/2 mg/kg xylazine/2 mg/kg acepromazine (KXA) solution diluted with saline. Animals were placed into a custom harness with the transplanted eye (left eye) oriented toward imaging probe. Animals received continuous oxygen through a breathing mask. Supplemental anesthesia with isoflurane at 0.5–2% was employed as needed. Multiple B-scans and fundus images were obtained from different positions to determine graft location, quality of engraftment and state of post-surgery gross retinal architecture. Rats exhibiting excess surgical trauma (e.g., choroid damage, optic nerve damage), transplant misplacement (epiretinal, choroid) or corneal opacity/lens cataract were eliminated from the study.

### Optokinetic testing

Every month, at 1–5 months post-surgery, the visual acuity of RD nude rats (transplanted, sham surgery, and non-surgery AMC controls) was measured by recording optomotor responses to a virtual cylinder with alternating black and white vertical stripes at 6 different spatial frequencies (Optomotry, Cerebral Mechanics, Alberta, Canada) as previously described [7]. Testers were unaware of rats’ assignment to experimental groups. The number of rats was variable at different time points due to rat mortality and availability of testers due to Covid (1 month post-surgery: transplants n = 4; sham + AMC n = 3; 2 months post-surgery: transplants n = 4; sham + AMC n = 9; 3 months post-surgery: transplants n = 5; sham + AMC n = 3; 4 months post-surgery: transplants n = 3; sham + AMC n = 2; 5 months of surgery: transplants n = 3; sham + AMC n = 3). Rats were dark-adapted for at least 1 h prior to testing. The best visual acuity of two same-day tests was used for analysis. Video recordings were evaluated off-line by two independent observers blinded to the experimental condition. If there was a discrepancy between the two observers, videos were re-analyzed by a third observer, and data discussed before giving a final score (prior to decoding the experimental condition). Because the numbers of rats for sham and AMC were low, and there was no significant difference between them, both sham and AMC were combined as control for statistical analysis.

### Superior colliculus (SC) electrophysiological recording

Visual responses from the SC were recorded as previously described after overnight dark adaptation [7]. Two immunosuppressed transplanted rats (age 7.0–7.4 months, 5.8–6.3 months post-surgery), 3 sham surgeries (age 7.5–8.4 months, 6.2–7 months post-surgery), and 3 age-matched controls (age 7.9–9.6 months) were recorded in the SC. Three transplanted rats (age 6.6–7.5 months, 5.4–6.1 months post-surgery) and 1 sham surgery rat (age 7.7 months, 6.5 months post-surgery) died before SC recording because of a heart attack in response to intraperitoneal Ketamine/Xylazine injection (dosages see Transplantation Surgery section). During recording, the tester was blinded to the group allocation of the animals. Multi-unit electrical responses from 50 to 70 locations on the SC surface approximately 200–400 µm apart, using a tungsten microelectrode (0.5 MΩ impedance; MicroProbe, Inc., Carlsbad, CA). At each location, light stimuli (20 ms, + 0.58 to − 6.13 log cd/m^2^) were delivered approximately 10-times at 10-s-intervals. When responses were found, the intensity of light stimuli was reduced until there was no response in order to determine the response threshold. Responses to the strongest light stimuli (stimulus level 0.58 log cd/m^2^) were quantified into a map over the area of the SC. All spikes occurring 30 ms to 210 ms after the onset of the photic stimulus were counted. Spike counts and locations of responses were analyzed using a custom MATLAB program (Mathworks, Natick, MA).

### LCMS/MS

Tacrolimus blood levels of a 90-day release 20 mg dose (specified by manufacturer to release 20 mg TAC in 90 days) were monitored via LCMS in a non-surgery dosing group to assess the drug pharmacodynamic profile.

#### Solid phase extraction

Isolation of tacrolimus from whole blood samples was performed by solid phase extraction methods detailed by [32]. Tacrolimus was isolated from 100 µl whole blood samples collected across 5 timepoints (approximately every 1.5 weeks) over the 90-day dosing period via solid phase extraction. Proteins were precipitated from thawed samples previously stored at − 80 °C. Whole blood samples were thawed in a 37 °C water bath and 50 µl of whole blood sample was combined with 250 µl D.I. water, 250 µl 0.2 M zinc chloride solution and 500 µl HPLC grade methanol (Sigma Aldrich, St. Louis, MO, USA). The solution was heated in a 37 °C water bath and simultaneously vortexed by sonication for 30 min, followed by centrifugation at 13,200 rpm for 15 min at 4 °C [32]. The resulting supernatant was added to 1 ml C18 HPLC grade column (Agilent Technologies, Santa Clara, CA, USA) preconditioned with 1 ml D.I. water followed by 1 ml HPLC grade methanol (Sigma) and washed using closed vacuum system air pump via solid phase extraction manifold (Supelco, Bellefonte, PA, USA). 1 ml pure HPLC grade acetonitrile was used as elution solvent. Standard reference solutions from concentrations of 1, 5, 10, 25, 50 and 100 ng/ml were prepared in 200ul of 80% methanol, 20% D.I. water with HPLC grade tacrolimus solution and a 2.5 ng/ml internal standard of C13 deuterated tacrolimus standard (Cerilliant, Round Rock, TX, USA). Extract was air dried and resuspended in 50% HPLC grade methanol into 1 ml vials and analyzed via time-of-flight mass spectroscopy, via the Triple Quad (Waters, Milford, MA, USA).

### Dosing group (pilot immunosuppressant dosing efficacy) (Table 1)

An overview of the different dosing regimen is provided in Table 1. TAC concentration in whole blood samples from 5 equidistant timepoints across 90-day dosing period were analyzed. TAC concentrations in each sample were calculated calibrated to their individual spectrometer area under curve values in reference to pure standard solutions across a given concentration range within the same run (0–100 ng/ml).

### Flow cytometry

Whole blood was collected from select *non-nude transplant IS* rats for lymphocyte immunophenotyping approximately every 4 weeks. Whole blood was collected, and lymphocytes isolated using RBC lysis buffer 1× (Biolegend, San Diego, CA). Lymphocytes were stained for the following markers with the listed antibodies and fluorophores: CD45RA+ B-cells APC, CD3+ T-cells FITC, CD4+ T-helper cells PE-Cy7, CD8α+ T-cytotoxic cells PE, BV421, CD11bc+ myeloid cells, PE-Cy7 and CD161+ NK cells with APC and Zombie viability dye with BV421 (each conjugated antibody and fluorophore used for cytometric analysis were also obtained from Biolegend; San Diego, CA USA) (see Supplemental Table S3). Cells were counted (at 10–30 k events) using the BD Fortessa X20 flow cytometer (BD Biosciences, Franklin Lane NJ, USA).

### ELISA

Plasma (35–100 ul) extracted from whole blood (100–150 ul) collected at 1–2 days and 3–4-weeks post-surgery spanning 4 cohorts, was evaluated for presence of inflammatory cytokine IFN-γ using an IFN-γ ELISA panel (cat. # 439007, Biolegend) and at 1 and 7 weeks post-surgery with IL-2 cytokine panel (cat. # ERA26RB, Thermofisher, Irwindale, CA) respectively.

The samples were diluted 1:2 and split into 3 biological replicates. Absorbance values were obtained using Bio-Tek Synergy HTX Spectrophotometer (Agilent). A standard curve was generated at 450 nm and serum concentrations of each respective cytokine. Concentrations of IFN-γ and IL-2 collected from serum of surgery animal at designated time points was determined by interpolation of absorbance values relative to standard.

### Tissue processing

At the end of the study (see Tables 1, 2, 3, 4), animals were either injected by an overdose of Ketamine/Xylazine (100 mg/kg Ketamine/ 20 mg/kg Xylazine) or euthanized by CO_2_ exposure followed by cervical dislocation. Eyes from animals in each experimental study category were obtained for histological analysis at study endpoint after superior colliculus recording (followed by ketamine/Xylazine overdose) via trans cardiac perfusion with 0.9% saline followed by 4% paraformaldehyde (PFA). Upon premature expiration (ketamine/xylazine—isoflurane induced cardiac/respiratory arrest), eyes were drop/immersion fixed in 4% PFA).

Eyes from selected animals of control/Sham/Transplant and no IS groups were immersion fixed in 4% PFA following CO_2_ euthanasia followed by cervical dislocation. In addition to the study elimination criteria described above in the OCT imaging section, sham surgeries and age matched controls constituted this type of tissue fixation.

Eyes were removed and an opening cut into cornea to facilitate complete penetration of fixative (overnight at 4 °C). After washing with 0.1 N Na-phosphate buffer, eye cups were dissected, transferred to 30% sucrose overnight and frozen down into O.C.T (optimal cutting temperature embedding medium, Fisher Scientific) blocks in isopentane on dry ice. 10 µm histological sections were prepared via cryosection at 3 sections per slide.

### H&E staining

Hematoxylin and eosin (H&E) staining was used to visualize the morphological structure of the retina. Details of protocol are described in Supplemental Methods. Slides with stained sections were then imaged with light microscopy at 10 × and 20 × magnification.

### Immunofluorescence staining

Slides selected for immunofluorescence staining underwent antigen retrieval with Histo VTOne (Nacalai USA, Inc. San Diego CA, USA) at 70 °C for 30 min followed by 3 ten-min washes in 1 × phosphate buffered saline (PBS) solution. Sections were isolated with a PAP pen and underwent blocking for 30 min with 1% PBS BSA, 20% donkey serum and 0.3% Triton-X100. This was followed by incubation with primary antibody solutions overnight in a humid chamber at 4 °C (see Supplemental Table S2) prepared in dilution buffer composed of 1% PBS/BSA and 20% normal donkey serum. After washing, sections were then incubated with secondary antibody solutions containing defined fluorophores for 30–60 min at room temperature in a covered chamber protected from light, followed by 30-min incubation protected from light with 4,6 diamindino-2-phenylindole (DAPI) at 50 µg/ml or 20 µg/ml Hoechst solution at room temperature. Slides were mounted with DAPI containing medium (Vector Labs, Newark CA, USA), cover slipped with nail polish (Electron Microscopy Sciences) and left to dry before cold storage and subsequent imaging. Immunofluorescence slides were imaged by a Zeiss LSM 700, LSM 900 (Oberkochen, Germany) and Leica SP8 (Leica Microsystems, Wetzlar, Germany) respectively.

### Histological analysis

Histological analysis of 10 µm retinal sections was performed using immunofluorescence antibody panels characterizing donor RO graft cells and infiltrating host immune cell populations at the transplant site, endogenous host retinal structures, and markers of functional phototransduction in relation to host and donor photoreceptors. Quantitation of immune cell subtypes and donor cells was performed using FIJI ImageJ (Version 1.54 h) and Adobe Photoshop Version 25.7 (Adobe).

Spatial parameters of graft area were outlined and measured in µm^2^ in Photoshop. Cell types identified by immune histochemical markers were counted using Image J within the designated graft area. Iba1/CD68 double positive cells were distinguished by morphology ameboid, non-activated or ramified, rounded activated morphology with prominent soma. Macrophages were further designated as either CD68+/Iba1+ or CD45+^hi^/Iba1+ with ramified morphology.

### Statistical analysis

Graphpad Prism 8 and 10 was used for statistical analysis. For the organoid drug treatment, a one-way ANOVA was used to compare experimental groups.

Welch’s t-test was used to compare the effect of TAC on B-cell and T-cell populations in the pilot dosing group. Ordinary one-way ANOVA with Dunnet multiple comparisons was used in analysis and comparison of *non-nude transplant IS* group lymphocyte populations to *non-nude AMC* groups at multiple timepoints throughout IS treatment, as well as analysis of cytokine levels between immune competent surgery and non-surgery groups.

Rats (either sex) were randomized into age-matched non-surgery, sham, and transplant cohorts. Experimenters were blinded to the condition of the animal. For all statistical analyses, the significance level was calculated in Graphpad Prism software (Graphpad Software LLC, La Jolla, CA) with paired and unpaired two-tailed t-tests using mean ± SEM. Level of significance was set at 0.05.

## Results

### RO specifications (Fig. 1a)

For in-process acceptance criteria, immunostaining was performed on horseshoe-like, adherent reticular striated structures that were cut out for continued 3D culture as RO prospective structure. Immunocytochemistry for eye field markers (Otx2 and Chx10) confirmed that these structures have an expression profile of developing human retina (data not shown). Figure 1a shows our final specifications for 3 GMP-compatible RO release lots at day of differentiation 55. Average viability was close to 90% (Fig. 1a–a1) as determined by trypan blue exclusion. No expression of the stem cell marker OCT4 was detected in qPCR analysis (Fig. 1a–a2), OTX2 expression in ROs was around 80% as determined by flow cytometry (Fig. 1a–a3), and almost all RO cells expressed the marker RAX as determined by immunohistochemistry (Fig. 1a–a4), flow cytometry and qPCR (data not shown).

### ROs are minimally immunogenic to sensitized human immune cells in vitro (Fig. 1b–e)

Human PBMCs exposed to both irradiated human fibroblasts and CSC14 derived ROs were evaluated for their capacity to elicit immune cell proliferation and immunogenicity. CSC14-derived ROs (60, 54, 49 days of differentiation) were co-cultured with sensitized human immune cells to test allogeneic immune response. MHC II expression and capacity to elicit immune cell proliferation as indicated by KI67+ cells were measured by flow cytometry as affirmative indicators of allorecognition, antigen presentation and expansion of effector cells in RO/human PBMC coculture. In Fig. 1b, the Ki67 gating of CD45+ CD3+ cells were determined by using antibody isotype controls. Ki67 High and Ki67 Low populations were observed. T-cells stimulated by beads and irradiated fibroblasts were highly proliferative. T-cells cultured with ROs were weakly stimulatory and were mostly found within the Ki67 Low gate (data not shown). Figure 1c shows representative flow plots demonstrating MHCII staining and gating. The three overlays are replicates for each respective lots and the green histogram represents the isotype control used for gating. In Fig. 1d, the percentage of live CD45+ cells that express MHC-II was determined and plotted for each condition. There was a significant difference (*p* < 0.0012) in MHC-II expression between the cells cultured with αCD3 microbeads or retina organoids. Figure 1e shows significantly reduced MHC-I expression in ROs compared to CSC-14 hESCs and irradiated fibroblasts. This data indicates that retina organoids are not likely to induce an immune response.

### Organoid drug experiments (FLIM and qPCR) (Fig. 2)

As shown in Fig. 2, we investigated the effects of mycophenolic acid (MPA), which is a hydrolyzed product of MMF, and TAC on RO development. Two sets of ROs (Set 61: start day 68; Set 52: start day 87) were treated with (1) 0.5 ug/mL MPA, (2) 3 ng/mL TAC, (3) a combination of 0.5ug/mL MPA and 3 ng/mL TAC, and (4) non-treated controls. The treated organoids were imaged using fluorescent lifetime imaging at 1 week (day 75 and day 94, respectively) and 4 weeks (day 95 and day 115, respectively) post-treatment to assess the free and bound nicotinamide adenine dinucleotide (NADH) ratio, indicative of their metabolic state.

Our results revealed that within the same set and same imaging timepoint, drug-treated groups showed no significant differences compared to the control group in terms of the free and bound NADH ratio (Fig. 2a, b, Supplemental Table S4). Figure 2c shows that the imaged control ROs fall within the ranges of previously investigated ROs [5] in terms of their free/bound NADH ratio at the imaged time points. This suggests that the immunosuppressant drugs MPA and TAC had minimal effects on the development of retinal organoids in vitro. The qPCR results revealed that TAC and MPA treated samples showed similar gene expression level as control. The combined treated sample showed a discernable fold change in downregulation of CRX and NRL compared to the other three groups (Fig. 2d).

### Dosing group establishes working therapeutic drug dosages and reveals the relationship between pharmacodynamic profile and immunosuppressive effect (Fig. 3)

**Fig. 3:**
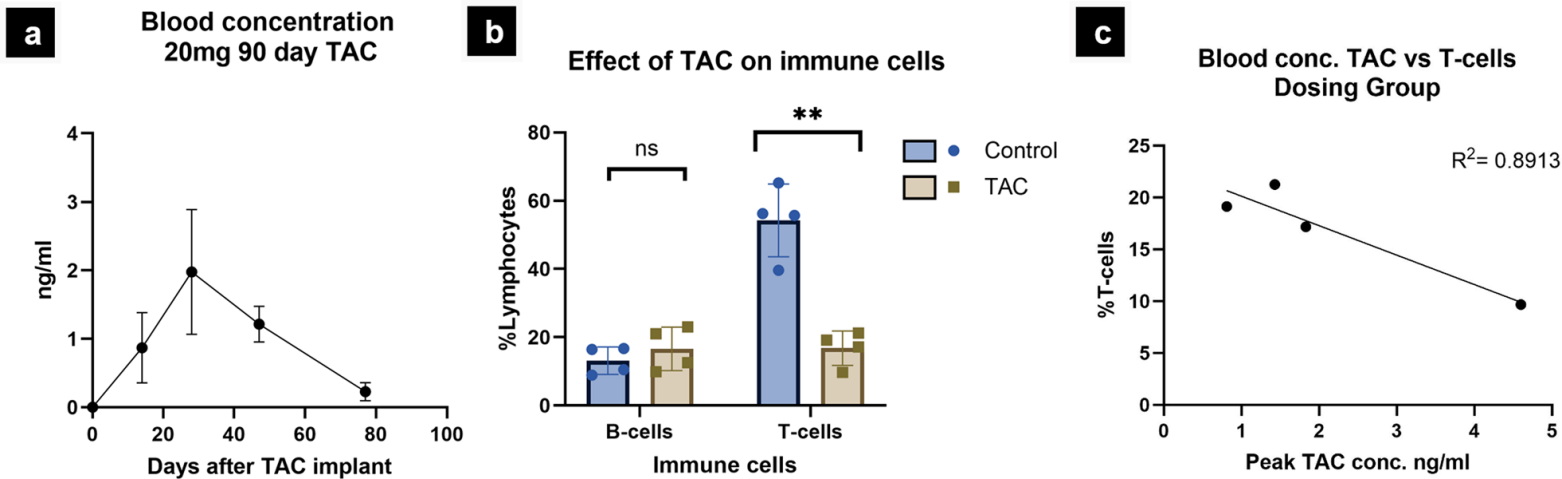
Therapeutic drug monitoring of tacrolimus 20 mg 90-day release absorption and elimination profiles. a Average group tacrolimus blood concentrations across 77 day treatment period. b Comparison of representative B and T immune cell populations between age matched non treated and dosing group RD rats after 20 mg 90-day tacrolimus implant (70–77 days post-implant). c Relationship between observed peak blood concentrations of tacrolimus and measured T-cell populations at 70–77 days of dosing

Initial *non-nude transplant IS* cohorts were administered stepwise increasing doses. Regimens 1–3 (Tables 1, 2), resulted in poor graft survival while regimen 4 (20 mg 90-day release TAC pellet) resulted in more prolonged survival. Then in depth analysis of drug distribution and effect on immune cell populations was performed in immunocompetent RD rats. for which a non-surgery dosing cohort consisting of 2 males and 2 females was recruited. LCMS/MS analysis of dosing group blood samples determined that administration of 20 mg 90-day release TAC pellet resulted in peak blood concentrations of 0.8–4.6 ng/ml occurring between day 25–55 in the treatment course (Fig. 3a). There was individual variability in magnitudes across sexes (see Supplemental Fig. S1).

Using flow cytometry based immune phenotyping, the suppressive effect of a 20 mg 90-day release TAC implant was evaluated near the dosing endpoint (day 70 for females, or day 77 for males). Figure 3b measured T-cell and B-cell populations of immunosuppressant treated versus non-treated age-matched RD rats. Figure 3c evaluates the relationship between given dose, resulting blood concentration and degree of T-cell suppression for individuals in our dosing group. The magnitude of T-cell suppression was positively associated with high peak blood concentration values of TAC (Correlation coefficient 0.89).

However immune suppression (specifically TAC) had a negative effect on responses to Ketamine anesthesia. 39/92 immunosuppressed rats prematurely expired due to cardiac arrest after injection with Ketamine/Xylazine for anesthesia, transplant surgery, optical coherence tomography or superior colliculus recording at dosages that would not cause an issue with non-immunosuppressed rats.

### Long-term graft tolerance in-situ has distinct morphology compared to acute rejection as determined by optical coherence tomography (OCT) (Fig. 4)

**Fig. 4:**
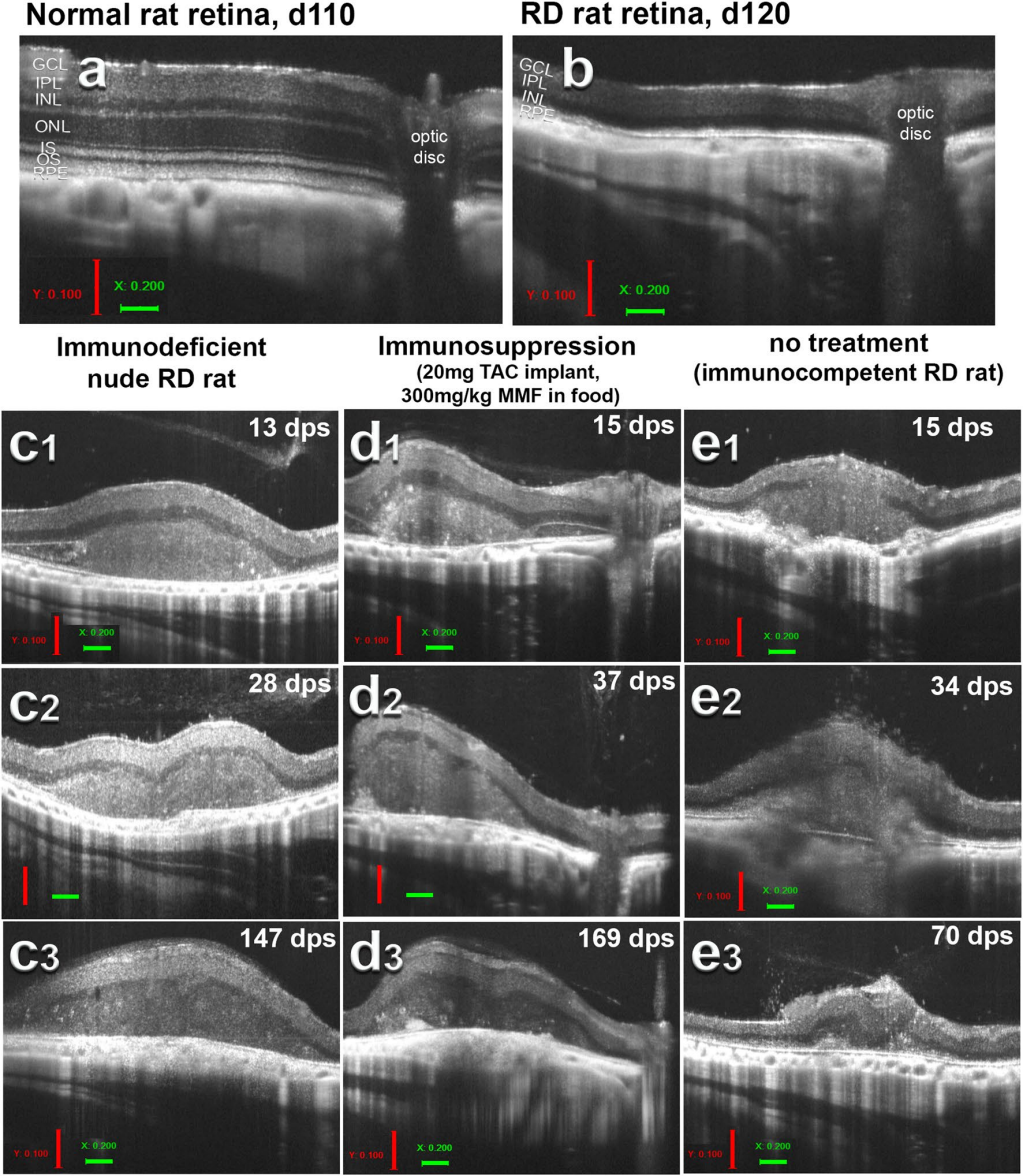
Optical coherence tomography (OCT) imaging. a and b OCT B-scans comparing approximately age-matched, a wild-type (WT) d110 and b retinal degenerate (RD) d120 retina. c–e OCT B-scans of retinal organoid tissue transplants in RD rats across treatment and immunocompetence modalities from initial scan, approximately 2 weeks post-surgery (c1-e1), up to 1-month post-surgery (c2–e2), up to final scan near study endpoint. Since the position of the transplant in the eye was variable between surgeries, the optic disc can only be seen in (d1–d3). c1–c3 Transplant to immunodeficient RD rat (*Nude TP*); d1–d3 transplant to immunocompetent immunosuppressed RD rat (*NN TP IS*), e1–e3 transplant to immunocompetent non-immunosuppressed RD rat (*TP NO IS*). Note graft size and integrity is maintained throughout study/observation period for both *nude TP* and *TP IS*. Successful immunosuppression mirrors long term graft survival seen in athymic rats with absent T-cells, whereas *NN TP NO IS* graft is besieged by immune cells at day 34, demonstrated by fuzzy graft appearance and reflective speckles in vitreous (e2). Faced with a competent non-suppressed immune response, this graft is nearly completely consumed by day 70 (e3). Vertical scale bars (red): 100 µm; horizontal scale bars (green): 200 µm

OCT was used for in vivo imaging of tissue graft and retinal structures. Initial imaging was performed 2 weeks after transplantation to evaluate graft placement and assess any damage to surrounding ocular tissues (Fig. 4c1, d1, e1). Retina of transplanted RD rats were evaluated for signs of ectopic graft placement (epiretinal or otherwise), damage to optic nerve, damage to choroid, severe retinal detachment, cataract, or visible acute rejection such as highly reflective speckles in vitreous accompanied by a ‘fuzzy’ appearance to graft and surrounding host tissues indicative of probable immune cell infiltrate. Makabe et al. [19] have similarly reported visibility of microglia as hyperreflective speckles of distinct and predicable morphology in OCT scans of RD animal models. Hyperreflective artifacts were observed in B-scans (the topographical 2D scan taken along the surface of the retina) of rejected transplants (Fig. 4e2). Nevertheless, any of these retinal aberrations, individually or in combination, were grounds for elimination of graft recipient from downstream optokinetic and electrophysiological assessment in the study. In the context of immune competent graft recipients, evidence of acute rejection and immune cell proliferation via reflective speckles and fuzzy graft site observed in OCT B-scans (Fig. 4e2) provided an unexpectedly novel initial qualitative indicator of acute rejection that was subsequently positively corroborated with immunohistochemistry results.

For both immune competent and deficient graft recipients, initial 2-week post-surgery OCT retina scans were followed by subsequent scans taken at intervals of 1–2 months throughout duration of the study wherein multiple timepoints allow monitoring of graft volume and location (Fig. 4c2, d2, e2). Transplant location in relation to optic disk was variable between experiments. Sham surgeries and non-surgery age matched controls (*non-nude AMC*) only received an initial OCT.

RO grafts in both nude (n = 8) and non-nude successfully immunosuppressed (n = 8) recipient cohorts alike maintained a consistent graft area and topographical location throughout the observation period. Of long term (2.5–6-month) surviving grafts, 50% (4/8) of nude transplant group transplants retained > 90% graft area, with 75% (3/4) of this number increasing in area by study endpoint. 43% (4/8) long-term surviving *non-nude transplant IS* group transplants retained > 90% graft area with 50% (2/4) of this proportion seeing an increase in graft area at study endpoint. Hence in vivo, among the IS treatment group, the absence of hyper-reflective rejection morphology and longitudinal maintenance of graft tissue area across subsequent scans demonstrated successful prevention of graft rejection delivered by a minimum therapeutic threshold immunosuppressive dose of tacrolimus and/or MMF treatment.

### Successful tacrolimus mediated T-cell suppression results in global attenuation of adaptive and innate immune responses (Figs. 5, 6)

**Fig. 5:**
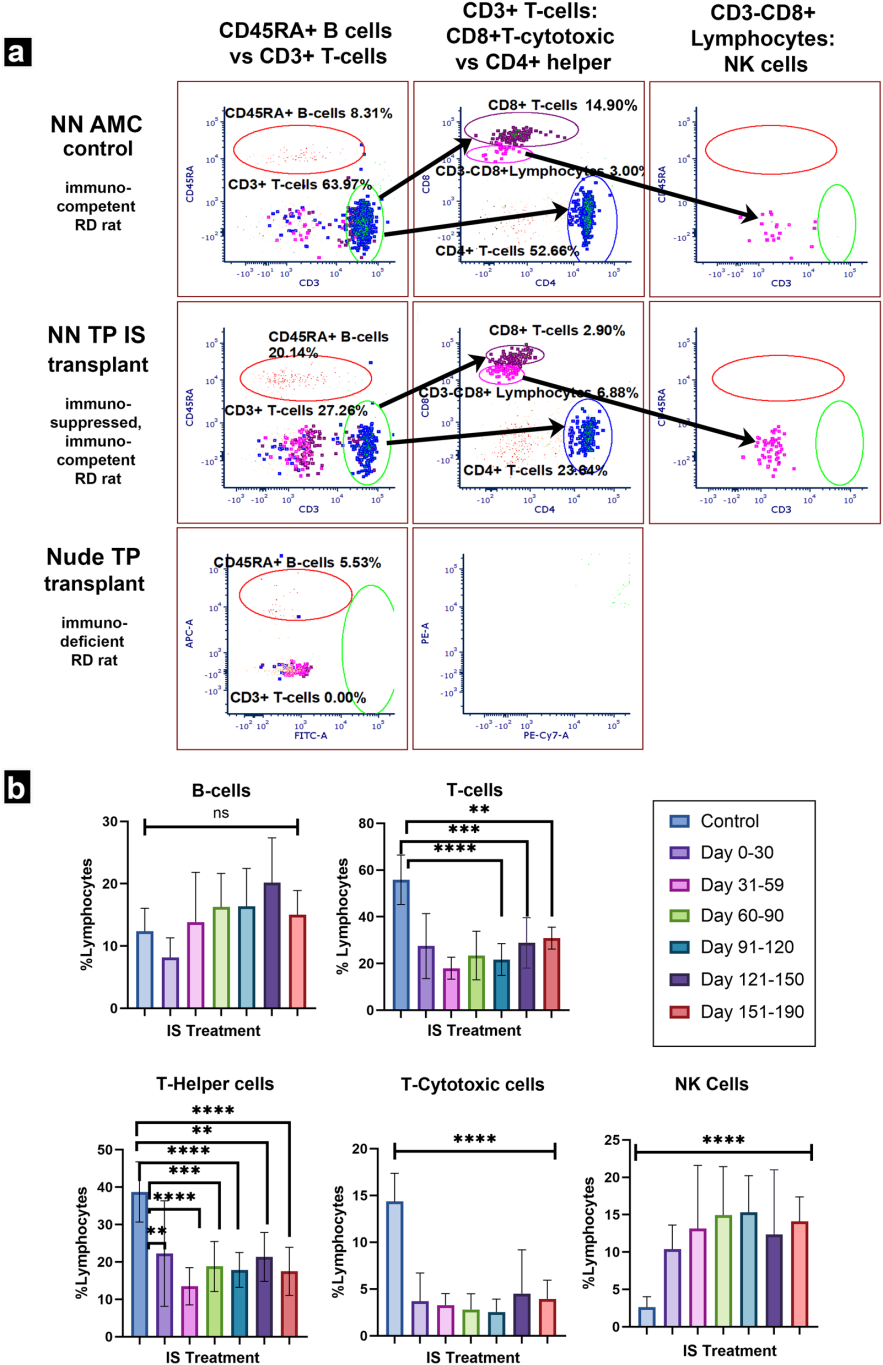
Immune phenotyping. a Representative flow plots of standard immune phenotyping gate scheme and its application to phenotyping of non-surgery untreated immunocompetent control (*NN AMC*) and IS treated (*NN TP IS*), immune competent transplanted RD rats in comparison to transplanted immunodeficient (nude/*foxn1−/−*) rat (*Nude TP*). b Key lymphocyte populations in *non-nude TP IS* group over treatment course. With dosing regimens #5, 20 mg TAC+ 150 mg MMF and #6, 20 mg TAC and 300 mg/kg, respectively. Treatment resulted in substantial suppression of CD3+ T-cell populations, expansion of CD3−CD8+ natural killer lymphocytes. B-cells undergo suppression in the initial 0–30-day time interval of treatment, followed by subsequent trend of expansion thereafter. Statistical analyses were performed using ordinary one-way ANOVA with Dunnet multiple comparisons. The statistical significance of each lymphocyte subpopulation census at each treatment timepoint is obtained by comparison to representative mean lymphocyte subpopulations from immunocompetent non-treatment, non-surgery age matched littermates

**Fig. 6:**
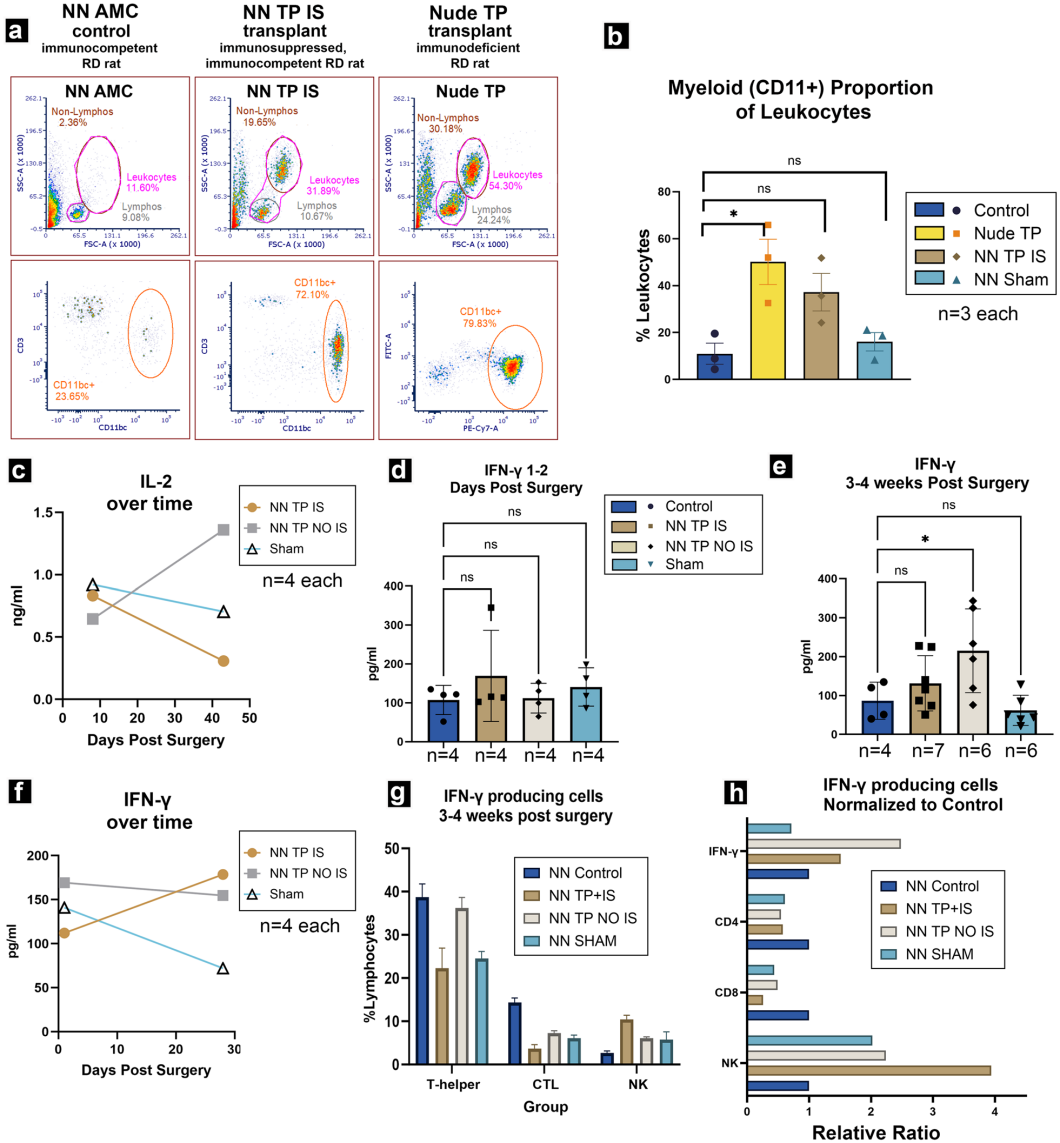
Flow analysis of immune cells; and ELISA of IL-2 and IFN-γ production. a Representative flow plots of leukocyte distributions of (from left to right) (1) non surgery, non-IS treatment control (*NN AMC*); (2) IS treated RO transplant recipient (*NN TP IS*); and 3) *foxn1−/− *mutant RO transplant recipient (*Nude TP*) RD rats. Uppermost row isolates lymphocyte from non-lymphocyte proportions of leukocyte population of each, based on characteristic forward and side scatter distribution. Where leukocytes are further distinguished into lymphocytes (“lymphos”) and non-lymphocyte leukocytes (“non-lymphos”). Lower row quantifies CD11bc+ myeloid populations from ‘non-lympho’ proportion of leukocytes from each respective immune status and treatment category. b Mean population profile of myeloid proportion of leukocytes (CD11 bc +) across immune status and treatment categories. Note immunocompetent sham surgery category is included. n = 3 each category. Both immune deficient (*nude TP*) and *NN TP IS* group demonstrating significantly higher relative populations of myeloid CD11 b/c+ cells than immune competent control or sham groups. *TP NO IS* group not represented. c Serum IL-2 levels of single surgery cohort approximately 1 week (8 days) and 7 weeks (43 days) post-surgery, Surgery groups: *NN TP IS* n = 1, NN TP NO IS n = 2, and *NN Sham*, n = 2. Mean values are given where n > 1. d and e Comparison of serum IFN-γ levels across immunocompetent surgery modalities 1–2 days and 3–4 weeks post-surgery and in non-surgery, non-treatment age matched RD control group. 1–2 days post-surgery: N = 4 for each group. 3–4 weeks post-surgery, for each group *NN AMC* n = 4; *NN TP IS* n = 7; *NN TP NO IS* n = 6; *NN Sham* n = 6. Statistical analysis performed using ordinary one-way ANOVA with Dunnet multiple comparisons test. *TP NO IS* expressing greatest average IFN-γ. (though not statistically significant compared to *NN TP IS*). The sham group expressed the lowest overall IFN-γ levels; likely a result of homeostatic, immune modulatory rebound from the initial inflammation inciting event of invasive surgery, well within range of the competent immune response. f Serum IFN-γ levels from 1–2 days to 3–4 weeks post-surgery (n = 4 individuals from each surgery modality spanning 2–3 surgery cohorts each group measured at indicated time points). g Proportion of IFN-γ producing cells across surgery categories at 3–4 weeks post-surgery and h Representative proportions of IFN-γ producing immune cells across treatment categories of immune competent RD rats. e Table combines relative populations of IFN-γ producing cells normalized to non-surgery control. Across treatment categories, interrogates relationship between T-cell and NK populations upstream and serum IFN-γ levels downstream. X-axis values represent ratios of indicated categories compared to a normalized to immune competent, non -surgery control (*NN AMC*) representative of immune homeostatic levels

Administration of 20 mg TAC and 300 mg/kg MMF resulted in a consistent immunosuppressant efficacy profile, as determined by flow cytometry based immune phenotyping. Index of mean lymphocyte populations (n = 7) was generated from immune phenotyping of non-surgery, non-IS treatment immunocompetent RD rats (*non-nude AMC*). Month one of treatment saw a reduction of CD4+ T-helpers by 41%, and of CD8+ T-cytotoxic lymphocytes (CTLs) by 74% in proportion to *non-nude AMC*. *Non-nude transplant IS* rats maintained statistically significant proportions of T-cell suppression throughout the treatment course.

After 1 month of treatment, CD45RA+ B-cells were suppressed by 33% of *non-nude AMC*, followed by pattern of gradual expansion at subsequent timepoints thereafter. Interestingly, compared to *non-nude AMC*, CD3−CD8+ natural killer cells saw an average increase of 400% in treatment group (*non-nude transplant IS*) (Fig. 5).

In *non-nude transplant IS* group, this unexpected NK cell expansion was accompanied by an increase in the myeloid compartment (70% greater than age matched immune competent control). Interestingly, we have similarly observed expansion of myeloid compartment and macrophage population (CD11bc+ cells) among *foxn1−/−* athymic rats (78% greater than age matched immune competent control) (Fig. 6a, b). We propose this unexpected NK and myeloid cell expansion may be a type of immune cell proliferative compensatory response to natural or induced immunodeficiency.

To appreciate the effects of IS treatment, we comparatively measured levels of IL-2 and IFN-γ (two key proliferative and pro-inflammatory cytokines active in graft rejection and targets of calcineurin inhibition) across our surgery groups using ELISA assays.

Serum levels of IL-2 were measured at 8- and 43-days post-surgery in a single surgery cohort (Fig. 6c). Non-IS (*non-nude transplant NO IS*) treated graft recipients saw an increase in serum IL-2 from 8 to 43 days post-surgery, whereas both IS-treated and sham groups saw a relative decrease in average serum IL-2 from 8 to 43 days. Interestingly, in terms of absolute values, at the initial 8 days post-surgery (DPS) timepoint, sham surgery had highest IL-2 levels followed by *non-nude* transplant IS then *non-nude transplant NO IS* (Fig. 6c). However, at 43 DPS, *non-nude transplant NO IS* demonstrated the highest absolute IL-2 followed by sham, with *non-nude transplant IS* expressing absolute lowest levels of the proliferative cytokine (Fig. 6c).

At both 1–2 days and 3–4 weeks post-surgery, IS treatment substantially dampened serum IFN-γ levels compared to non-treated immunocompetent graft recipients (*non-nude transplant NO IS*) (Fig. 6d–f). This suppressive effect is particularly noteworthy for the *non-nude transplant IS* group despite (1) the presence of compound inflammation inciting incidents of surgery and xenograft transplantation combined and (2) a consistent trend of dramatic increase in populations of IFN-γ *secreting* NK cells and IFN-γ *stimulated* macrophages when compared to *non-nude AMC* counterparts (Fig. 6g, h).

Interestingly, in our *non-nude transplant NO IS* group, T-cells were in the low median range of the non-surgery non-IS control group (Data not shown). This was an unexpected finding, as we anticipated some degree of serum T-cell expansion reflecting the acute rejection concurrently observable in vivo with OCT and subsequently in IHC. Another unexpected immune phenotyping observation was found in our sham surgery group with the low median T-cell populations accompanied by the lowest IFN-γ levels amongst all surgery groups, including non-surgery control group (Fig. 6e).

### OKT and SC recording results (Fig. 7)

**Fig. 7:**
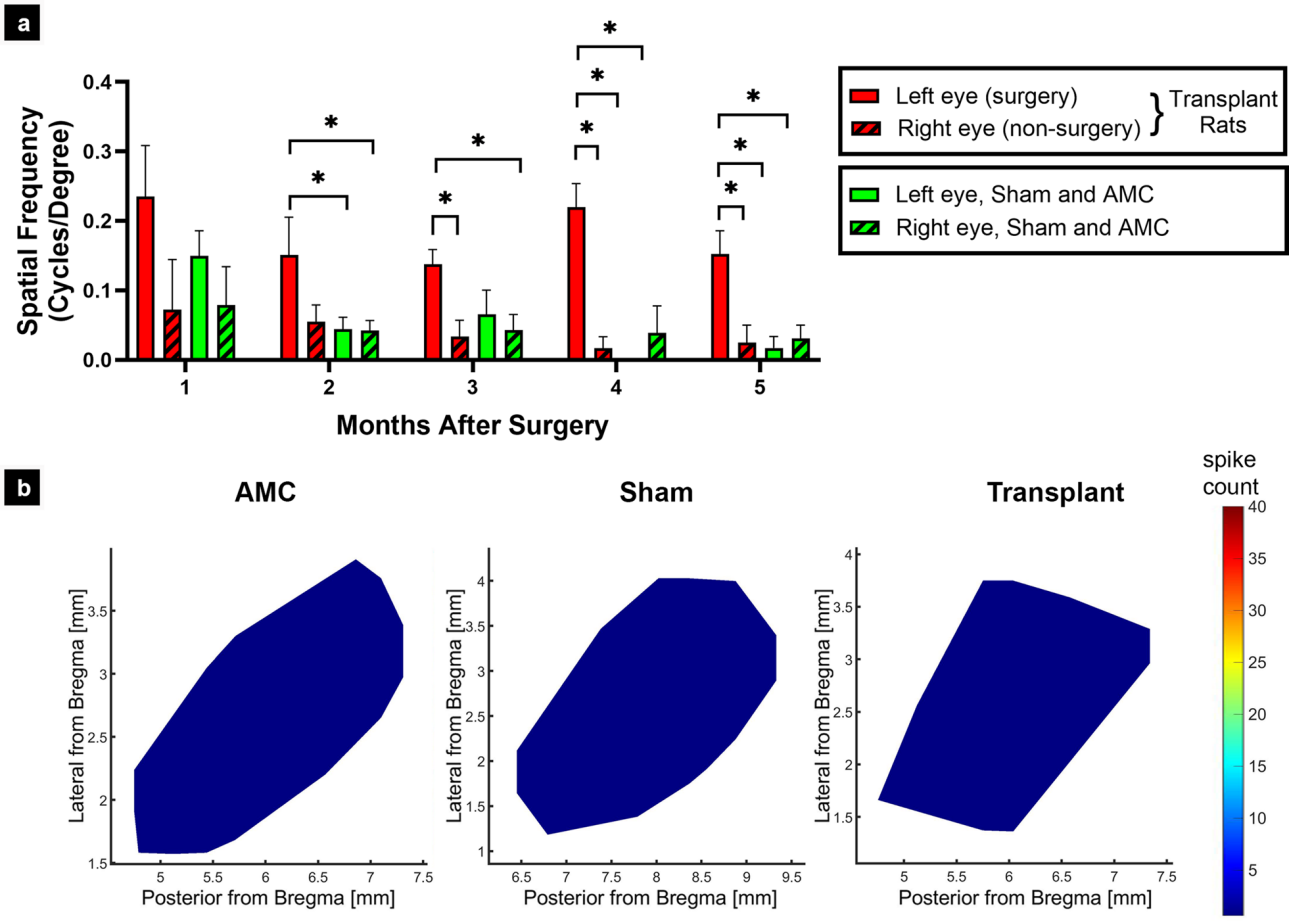
Functional testing of transplants to immunosuppressed RD rats. a Optokinetic testing of transplanted RD rats (red bars), compared with age-matched non-surgery RD controls (AMC) and sham surgeries (Data of both AMC and sham combined because of low N; green bars). Note that the transplants were always placed into the left eye at the age of 30–48 d. One month post-surgery: transplants n = 4; sham + AMC n = 3; 2 months post-surgery: transplants n = 4; sham + AMC n = 9; 3 months post-surgery: transplants n = 5; sham + AMC n = 3; 4 months post-surgery: transplants n = 3; sham + AMC n = 2; 5 months of surgery: transplants n = 3; sham + AMC n = 3. The number of experiments was lower for older ages as there was some mortality and rats were euthanized for immune cell phenotyping by flow cytometry and histology. b Example of SC recording maps of non-surgery AMC (age = 233 d), sham surgery (age = 225 d), and transplant (age = 223 d). No responses to light could be recorded

As shown in Fig. 7a, the RD AMC and sham showed degeneration with age when tested by OKT (number of rats is listed in the Fig. 7 legend). As measured by paired t-tests (between eyes of the same animals) and unpaired t-tests (between experimental groups), there was no significant difference between left eye (LE) and right eye (RE) in sham surgery and AMC groups (data not shown); thus, their data were combined as the control (sham and AMC). In addition, the right non-surgery eyes of the transplanted rats showed the same degree of visual acuity loss as non-surgery AMC and sham surgery rats. However, at 2–5 months post-surgery (MPS), significant improvement was observed in the transplanted eyes compared to the non-surgery right eyes of the same rats. This difference became larger at later time points (Fig. 7a). The improvement was also significant compared to control rats (non-surgery AMC and sham surgery) at 2–5 months post-surgery (*p* < 0.05; Fig. 7a).

### Superior colliculus recording results (Fig. 7b)

Two immunosuppressed transplanted rats, 3 sham surgeries (age 225–253 d, 187–211 d post-surgery), and 4 age-matched controls (age 237–289 d) were recorded in the superior colliculus (Fig. 7b) to detect light responses to a full light stimulus. However, no light responses could be recorded, even at the highest light intensity of + 0.58 log cd/m^2^. Furthermore, three transplanted rats and one sham rat (age 199–224 d, 161–182 d post-surgery) died before SC recording because of a heart attack in response to ketamine injection.

### Rejection versus tolerance at tissue and cell level (Figs. 8, 9)

**Fig. 8:**
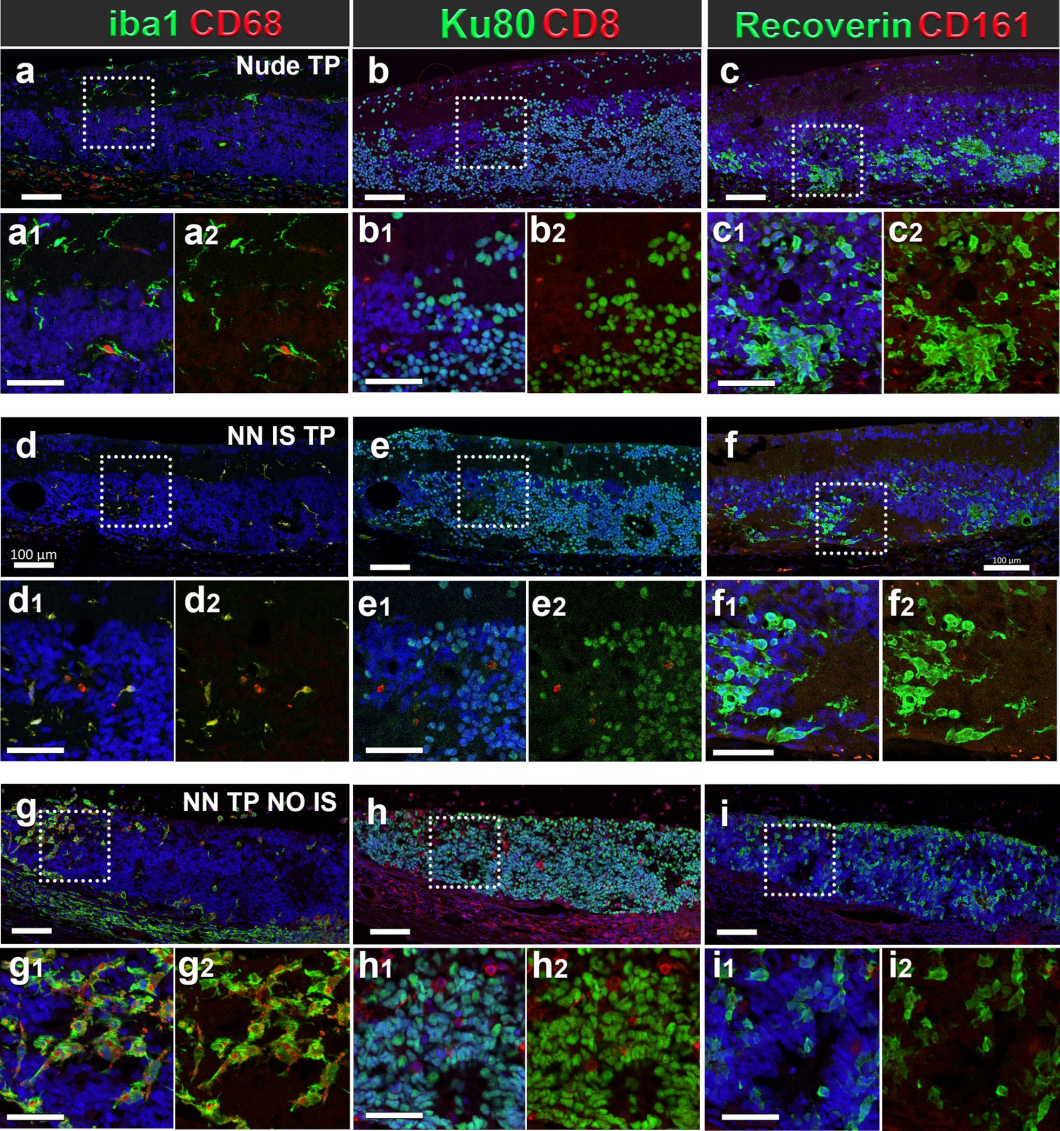
Immunohistochemical evaluation of graft outcomes across divergent immune competence modalities of a–c immunodeficient (*Nude TP*), d–f immune suppressed immune-competent (*NN TP IS*) and g–i non-immune suppressed immune-competent (*NN TP NO IS*) RD rats. IHC panel to investigate immune cell responses, identify graft cells and their photoreceptor identity. Segmentation of each stained section represents corresponding marker colocalization of a, d, g Iba 1 and CD68 (microglia/macrophage markers), b, e, h Ku80+ (RO graft cells of human origin) and CD8+ (CTLs), c, f, i Recoverin (calcium channel protein in photoreceptors and cone bipolar cells), CD161 (natural killer lymphocytes). Both *Nude TP* and *NN TP IS* long term survival transplants (6.7 and 6.3 months post-surgery) show similar distribution and density of ramified microglia (a, d) (wherein the only CD68 immunoreactivity was found in vascular endothelial cells of the choroid and retinal blood vessels), b, e dense aggregations of graft cells are found in ONL, while both IS and non-IS grafts are devoid of CD8+ T-cells. c, f Recoverin positive photoreceptors of graft origin are brightly fluorescent and often aggregate as dense mini rosettes. Conversely, host recoverin positive cone bipolar cells stain dimly and are found in the inner nuclear layer (INL). No CD161+ NK cells are found in the subretinal space of either *nude TP* or *NN TP IS* RD retina sections. g–i Xenograft Rejection resulting from no immune suppression: *NN TP NO IS*: Outcome of hESC-RO graft in immune competent RD rat 14 days post-surgery. g Graft immune cell infiltrate consisting of double positive Iba1+/CD68+ microglia with characteristic ameboid activated morphology and h abundant CD8+ T-cells among densely packed cells of RO tissue graft, indicated by Ku80 human nuclear protein staining (green). i Absence of positive CD161 reveals no natural killer lymphocytes present in this rejected graft. Cells staining brightly with recoverin indicate developing photoreceptors among transplanted RO cells. Dotted rectangles indicate areas of enlargement. Scale bars = 100 µm for the overviews, 50 µm for the enlargements

**Fig. 9:**
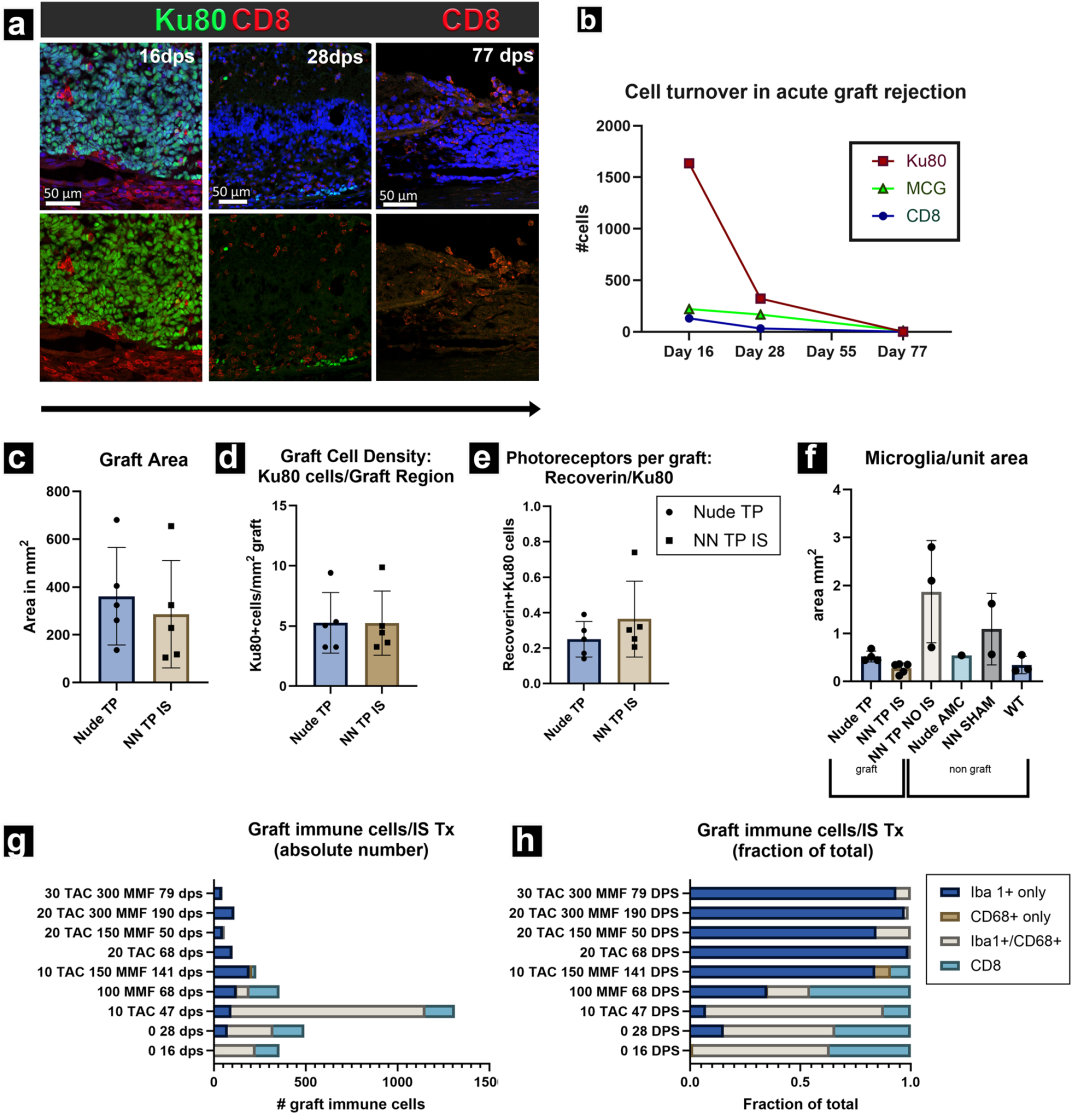
Quantitative analysis of immunofluorescence staining. a Timeline of acute rejection as observed in representative immunocompetent RO graft recipients. As early as 16 dps, graft is besieged by CD8+ T-cells. By day 28, graft tissue is functionally obliterated with only very few cells remaining. On day 77, only a hollowed-out space remains in place of graft, yet CD8+ T-cells remain in graft region. b Representative plot of absolute numbers of donor and key immune cell populations at defined points of acute rejection. c–f Key IHC metrics across immune status and treatment modalities. c Average graft area, d fraction of total immune cells in graft per corresponding IS regimen. e We determined the purity of graft cells by Recoverin+/Ku80+ cells. While there was no noteworthy variance in graft photoreceptor purity determined by between groups, average photoreceptor/graft density of 25% across all modalities is noteworthy. f Microglia graft density. *NN TP NO IS* contained the greatest density of microglia (by an order of magnitude) representative of rejection, whereas *NN TP IS* group displayed the lowest microglial density burden. g and h Absolute and fractional immune cell infiltrate composition profiles in the *NN TP IS* group. We evaluated graft immune cell density and composition per dosing gradient and type (mono and combination therapy) of IS treatment regimens. Y-axis represents an increasing gradient in dose efficacy which largely align with gradations in dose potency. Doses ≥ 20 mg 90-day release TAC represent successful graft tolerance, with graft rejection observed in lower subtherapeutic doses and 0 dose negative control

In immunodeficient graft recipients (*Nude TP*) (Fig. 8a–c), and in successfully immunosuppressed immunocompetent graft recipients (*non-nude transplant IS*) (Fig. 8d–f) graft tolerance was observed with no infiltrating T-cells, NK cells or peripheral macrophages at graft site. Microglia were present in immune tolerant grafts with a density at and adjacent to graft site comparable to immunodeficient, transplant tolerant counterparts (Fig. 8a2–c2, d2–f2).

In non-IS treated immunocompetent graft recipients (*non-nude transplant NO IS*) and cohorts with low dose, insufficient immunosuppression below therapeutic threshold (< 20 mg TAC monotherapy, MMF monotherapy, and < 20 TAC 150 MMF combination therapy), acute rejection was observed and characterized histologically by proliferative expansion and infiltration Iba1+ /CD68+ microglia with activated morphology and CD8+ T-cells. Microglia were identified by Iba1 positivity and ramified morphology. Non-reactive, resting microglia were qualitatively/quantitatively identified as Iba1+ /CD68-, ramified, with small soma and branching processes (Fig. 8a2, d2), whereas Iba1/CD68 double positivity accompanied by large, rounded soma with ameboid morphology were indicative of activated microglia (Fig. 8g2). Cells with CD68 marker alone were evaluated on their morphology, and location. Iba1/CD68/CD45^lo^ phenotype in ameboid immune cells was the most definitive confirmation of microglial identity (see Supplemental Fig. S2).

### CD8+ cytotoxic T-cells are necessary for acute RO graft rejection

CD8+ cytotoxic lymphocytes were the primary source graft rejection, responsible for directly engaging and killing graft cells.

Histologically, this was observed in the infiltration and expansion of donor specific cytotoxic lymphocytes at graft site. In early acute rejection (D 0–16), dense numbers of CD8+ and Iba1+/CD68+ cells are observed marginating along choroid, with many penetrating the graft site and engaging with donor cells (Figs. 8h, h1, h2, 9a1–a2). Mid to late acute rejection (D16–50) is characterized by more extensive cytotoxic lymphocyte penetration of donor tissue mass (Fig. 9a3–a4). Ultimately, most donor cells are destroyed at the end-stage acute rejection (D50–77) with residual cytotoxic lymphocytes, and microglia present to clean up (Fig. 9a5–a6). CD8+ cytotoxic lymphocytes are most abundant in early-mid acute rejection and appear to slowly decline as Ku80 graft cells are obliterated. Iba1+/CD68− microglia continue to proliferate at the graft site and persist in high density in mid and late acute rejection where virtually all donor cells have been obliterated (Fig. 9b). In rejected transplants with subtherapeutic, insufficient IS (< 10 mg TAC monotherapy up to 20 mg TAC+ 150 mg/kg MMF), the proportion and absolute numbers of CD8+ cytotoxic lymphocytes and microglia at graft site were directly correlated to gradations in potency of administered IS drugs in monotherapy and combination across dosing ranges (Fig. 9g, h).

Conversely, successfully IS treated rats demonstrated histologically absence of CD8+ cytotoxic lymphocytes (Fig. 8e). This directly corroborated with the degree of T-cell suppression observed with in vivo immune phenotyping at optimal working doses > 20 mg 90-day TAC at 0.74–3.17 mg/kg day. Immune-tolerated grafts (Fig. 8d) were associated with populations of ramified Iba1+/CD68− microglia of similar distribution and activation state as their immunodeficient transplant tolerant counterparts (Fig. 8a).

Figure 9c–e demonstrate comparable graft area, graft cell density and photoreceptors (as determined by histological analysis) between transplants to immunodeficient and immunocompetent rats with successful IS. Microglia density was low and comparable between the 2 groups whereas it was highest in the non-nude NO IS group (Fig. 9f).

### RO transplants in TP + IS group demonstrate comparable structural engraftment profiles to immunodeficient rats with improved visual acuity

**Fig. 10:**
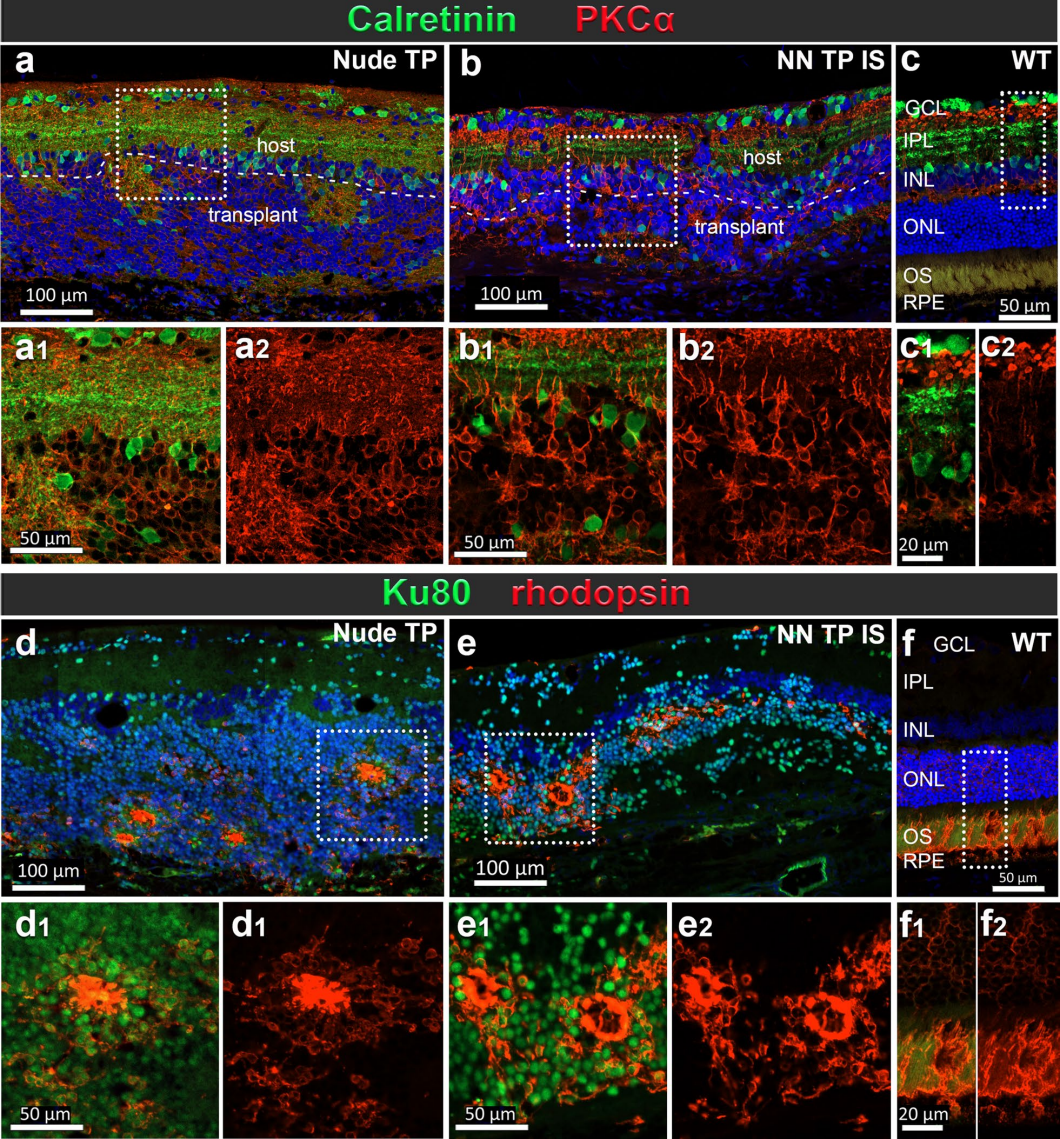
Transplanted photoreceptor sheets in both *Nude TP* and *NN TP IS* groups demonstrate similar engraftment within host retina architecture. **a**, **d** Transplant to nude RD rat (*Nude TP*). **b**, **e** Transplant to non-nude immunosuppressed RD rat (*NN TP IS*). **c**, **f** Normal rat retina (WT). **a**–**c** Calretinin positive (green) retinal ganglion cells and protein kinase C α (PKCα) positive (red) rod bipolar interneurons survive in both immunocompromised (**a**) and immune competent (**b**) retinal degenerate rats alike. However, in RD, these layers present with increasing levels of disorganization and destratification compared to non-RD wild-type (**c**). There is few Calretinin but a lot of PKCα staining in the transplant (**a**, **b**). **d**, **e** CSC14-derived RO transplants (labeled by human nuclear marker Ku80, green) contain mature rod photoreceptors expressing rhodopsin [40] which are organized in rosettes with outer segments in center, in contrast to the layered WT retina (**f**). Scale bars = 100 µm for the overviews, 50 and 20 µm for the enlargements

In wild-type retina, the 4 primary strata of phototransduction, from outer to innermost are retinal pigment epithelium (RPE), rod and cone photoreceptors (PRs), bipolar cells (BP) and retinal ganglion cells (RGCs) (Fig. 10c, f). Rod bipolar cells are identified with antibodies recognizing PKCα, with synaptic terminals close to the ganglion cell and in the inner plexiform layer [33, 34]. The initial photoreceptor loss of RP results in extensive remodeling and rewiring of bipolar interneurons to remaining, rapidly dwindling population of photoreceptors to compensate for lost transduction circuits. As PR degeneration outpaces compensatory remodeling of phototransduction circuits, the advanced disease is structurally characterized by increasing disorganization of interneuron layers and eventual collapse of the inner retina, leaving only RPE, Müller glia, bipolar and RGCs (Fig. 10a–c; Supplemental Fig. S3).

The subsequent diminution of retinal thickness and loss of structural integrity is observed with advanced RP in our AMC and sham groups, as well as areas distal to the graft in our transplant recipients.

**Fig. 11:**
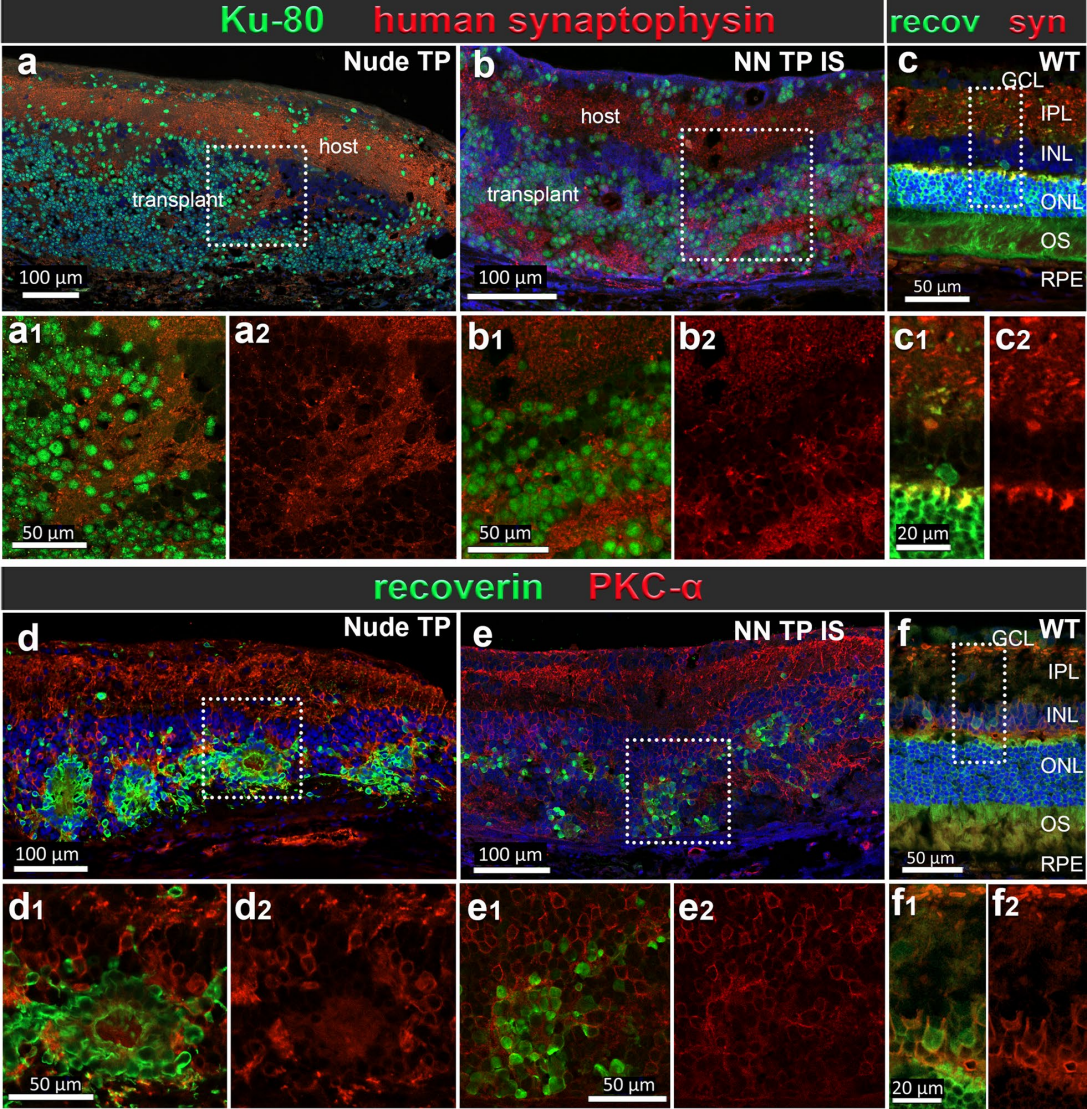
Transplant and retinal cell markers. **a**, **d** Transplant to nude RD rat (*Nude TP*). **b**, **e** Transplant to non-nude immunosuppressed RD rat (*NN TP IS*). **c**, **f** Normal rat retina (WT). **a**–**c** Expression and distribution of human synaptophysin (red) in transplants (immunoreactive for Ku80, green) in comparison to rat synaptophysin in healthy retina shows similar colocalization in RGC and inner plexiform layers, respectively. **a**, **b** Transplants demonstrate synaptophysin production and exocytosis (staining with antibody specific for human synaptophysin). **c** Normal rat retina, stained with antibody that recognized both human and rat synaptophysin, and recoverin antibody. Synaptophysin labels inner and outer plexiform layers. Recoverin is expressed in photoreceptors and cone bipolar cells. **d**–**f** Combination of recoverin (Photoreceptors [PRs], cone bipolar cells, green)—PKCα (rod bipolar cells, red), **d**, **e** Recoverin positive RO PRs integrate with host PKCα retinal bipolar interneurons. Densely packed rosettes (with outer segments facing inward toward lumen) are a frequently occurring morphology in photoreceptor transplants associated with improved visual acuity. **f** Normal WT retina. Scale bars = 100 µm for the overviews, 50 and 20 µm for the enlargements

**Fig. 12:**
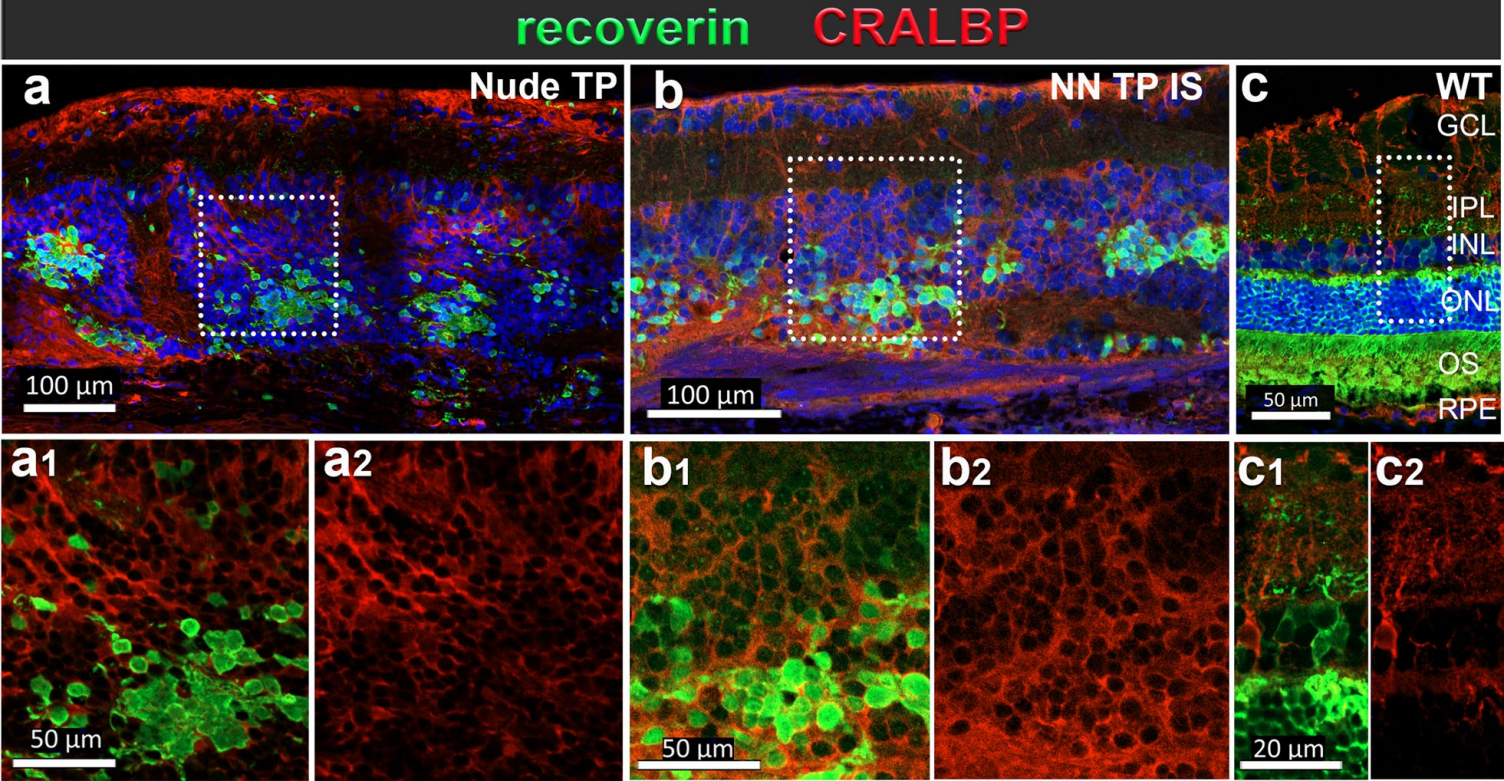
Photoreceptor and glial markers. Recoverin (photoreceptors, cone bipolar cells)—CRALBP (Müller cells and RPE): **a** Transplant to nude RD rat (*Nude TP*). **b** Transplant to non-nude immunosuppressed RD rat (*NN TP IS*). **c** Normal rat retina (WT). Scale bars = 100 µm for the overviews, 50 and 20 µm for the enlargements

Transplanted CSC14-hESC-derived retinal tissue sheets integrated within RD host retinal architecture. The host retina contained calretinin+ RGCs and amacrine cells (with three Calretinin+ strata in the inner plexiform layer) and PKCα+ rod bipolar cells overlaying the transplant (Fig. 10a, b). Few Calretinin + cells were found in the transplants, whereas many PKCα+ cells were observed. Transplanted RO sheets confirmed by Ku80 (human nuclei) staining contained rhodopsin-expressing rod photoreceptors, with strongly rhodopsin+ [40] staining outer segments in the center of rosettes (Fig. 10d, e). Furthermore, human synaptophysin (hSyn) colocalized with Ku80 positive cells exclusively in grafted regions but was absent in non-grafted regions, demonstrating RO grafted cells’ capacity for functional intersynaptic transmission and potential for interneuron signal transduction (Fig. 11a, b). Ku80 positive cells migrated into the host retina (Figs. 10d, e, 11a, b), and hSyn-positive transplant processes extended into the inner plexiform layer of the host (Fig. 11a, b). We observed close apposition between processes of recoverin+ donor photoreceptors, host bipolar interneurons and host/graft Müller glia (Figs. 11d, e, 12a, b).

In summation, this engraftment phenotype, characteristically observed in immune deficient (nude) RD rats is also shared by their immune competent counterparts (*non-nude transplant IS*) who have undergone successful immunosuppression.

### Adverse events

Rats in the dosing group demonstrated attenuated growth/weight gain profile compared to non-treated age matched controls. Several (39/92 = 42%) animals were lost from the study due to poor survival from anesthesia. Some died in pre-surgery (6/92) or post-surgery recovery (29/92) during the initial transplantation procedure, while others (14/92) died during recovery from anesthesia required for subsequent OCT procedures. Each anesthesia related death was characterized by cardiac/respiratory arrest, due to what we identified as the lethal interactions between calcineurin inhibitors (tacrolimus) and ketamine/xylazine, especially at successive doses required for maintenance of anesthesia during initial transplantation surgery, and repeated successive diagnostic procedures (OCT, SC). This introduced several issues, as premature deaths due to such adverse interactions negatively impacted the depth of our functional assessments the statistical breadth of our assessments of long-term dosing efficacy of our IS treatment while confounding the overall picture of their safety.

Other adverse events limiting the number of *non-nude transplant IS* from long term study included elimination by elective post-surgery euthanasia due to tissue damage (12/79), or corneal abrasion (4/79) procedure related accident (7/79), or otherwise unknown causes (9/79).

Two *non-nude transplant NO IS* group animals experiencing graft rejection (2/79) from subtherapeutic doses (confirmed by OCT) were removed from study for either histological analysis, LCMS and/or flow immune phenotyping training.

## Discussion

In several immunocompromised retinal degenerate animal models, we have demonstrated vision improvement by transplantation of hESC derived RO sheets [4, 7, 8] (review: [9]). Because immunosuppression is necessary for most of the transplantation treatments, our next step was to test the effect of immunosuppressants on ROs in vitro and in vivo (in an immunocompetent RD animal model). Similar to our previous work, before the transplantation, the ROs were specified by high viability (~ 90%), absence of stem cell markers, high purity (mainly photoreceptor progenitors), and minimal immunogenicity to sensitized human immune cells. In vitro experiments revealed that the immunosuppressant drugs MPA (a hydrolyzed product of MMF) and TAC had no significant effects on the development of ROs. In vivo tests showed that the immunosuppressant drugs MMF and TAC helped the ROs survive in immunocompetent RD host retinas for a long time by T-cell suppression; and induced vision improvement.

ROs are an invaluable resource for investigating retinal development, physiology and pathology toward development of potential treatments in retinal diseases.

In the defined context of tissue transplantation, wherein ROs are engineered and maintained for harvest upon defined standards of physiological maturity for transplantation in living subjects, clinical grade, standardizable levels of quality assurance across domains of identity, functionality, immunogenicity and reproducibility are required.

We accomplished with a reliably reproducible protocol for development of ROs whose genomic, cellular and tissue organizational functional metabolic authenticity we validated.

We then addressed potential graft safety of ROs with respect to immunogenicity. The mixed lymphocyte reaction assay of CSC14-hESC-derived ROs co-culture with sensitized human immune cells suggests low immunogenicity. We used human PBMCs and not rat PBMCs to mimic the future allograft situation in a prospective human patient. Of course, ROs derived from hESCs would elicit a strong response from rat (or mouse) PBMCs) as we have shown in our non-IS treated immunocompetent transplant group in vivo. We acknowledge, however, the inherent limitations of in vitro recapitulations of an embodied physiologically bound adaptive immune response and propose such outcomes an adequate preliminary predictive proxy of CSC14-derived ROs ability to elicit immune response in vivo in prospective human subjects.

The efficacy of any potential graft or cell replacement therapy is contingent upon host immune tolerance. TAC and MMF are clinical standard of care drugs with established safety and efficacy profiles routinely administered to promote tolerance of solid organ grafts in humans. Validation of their successful administration in previous stem cell xenograft tolerance animal studies [30] made them logical candidates in our preclinical study of retinal tissue grafts.

Individual variabilities in achievable TAC blood concentrations and subsequent immune suppressive effects at given dose is observed in human subjects and has often been attributed to genetic polymorphisms in second pass drug metabolizing enzyme cytochrome P450 [35, 36].

An in vitro exposure assay was used as a necessary preliminary measure to test the effect the two IS drugs at probable serum concentrations on the development or function of the ROs. The time points chosen for the drug treatment of the ROs were selected based on differentiation ages of ROs used for transplantation surgeries. Two lots of ROs were exposed to serum concentrations of IS drugs from day 68–115. This range of differentiation ages adequately represents the ages of ROs transplanted in the surgeries of this study. In previous publications [7, 37, 38], our group has emphasized the rationale and utility of transplanting photoreceptor (PR) precursors as opposed to terminally differentiated late-stage PRs. PR precursors are selected at this stage for their capacity to optimally engraft and their potential for functional maturation in vivo.

We acknowledge that direct exposure by way of immersion in culture media does not recapitulate and is not equivalent to any possible transient graft exposure to physiological serum concentrations of drugs via diffusion from peripheral circulation into the subretinal microenvironment. We reason that the diffusion and uptake profiles of direct immersion in culture favor optimal RO exposure to clinically relevant serum concentrations of immunosuppressant drugs whose outcomes may be reasonably extrapolated to a physiological environment.

Our previous experiments in RO-grafted nude rats revealed that absence of T-cells alone resulted in long term graft survival, across all ranges of functional rescue outcomes. On the surface, such results are intuitive, in agreement with first principles of adaptive immunology and provide foundational inductive basis for hypothesizing that targeted suppression of T-cell activity is the necessary component of achieving graft tolerance in immune competent individuals.

In our *non-nude transplant IS* group, the concurrent growth of each rat over time was conveniently coupled with the constant release rate of a TAC pellet and the administration of MMF chow at a fixed dose, manifesting in a gradual ramping down of dose potency (in mg/kg) throughout treatment. This mimicked the practice of weaning off immunosuppressant drug doses employed by clinicians in solid organ transplantation, where the highest doses of immune suppressant are administered throughout the critical period encompassing pre-surgery conditioning up to a defined time point post-transplantation (when the risk of acute rejection is greatest) to promote graft tolerance.

We determined the minimum therapeutic thresholds of TAC and MMF (20 mg TAC at 0.74–3.17 mg/kg day, MMF 300 mg/kg from 8 to 13.5 mg day) required to establish graft tolerance in an immunocompetent retinal degenerate rat model. We demonstrate that in immune-competent RD rats, targeted T-cell attenuation alone can reproduce an ideal immune histological profile similar to that observed in immunodeficient graft recipients without adaptive immune response, capable of facilitating survival and function of grafted photoreceptors. Conversely, acute RO graft rejection was exclusively observed in non-IS treated immunocompetent rats who maintained populations of functional, sensitized CD8+ and CD4+ effector cells not exposed to calcineurin inhibition.

In our non-nude IS group, the degree to which CD4 and CD8+ T-cell populations were suppressed upstream by TAC + MMF treatment regimens 4–7 (see Table 1) were directly reflected in attenuation of serum IL-2 and IFN-γ levels downstream. IS mediated attenuation of T-cells and serum levels of their primary proliferative and effector cytokines resulted in long term graft survival, absence of graft infiltrating T-cells, and a profile of non-reactive, graft agnostic microglia that mirrored long-term surviving grafts in immunocompromised *foxn1−/−* RD counterparts that have demonstrated both graft tolerance and functional improvement of visual acuity.

As IFN-γ is strongly implicated in activation of microglia, we propose its downregulation via absent T-cells is the inflammatory missing link responsible for the resting, agnostic microglia phenotype observed in immunocompromised*/foxn1* mutant *and* in successful IS treated RO-transplanted rats with substantially attenuated serum levels of both the cytokine and T-cells themselves.

The *non-nude sham* group expressed the lowest IFN-γ levels of all surgery groups. We speculate this may be a feature of an endogenous homeostatic immunosuppressive effect of immune competent sham surgery animals—a recovery profile that immune competent individuals would experience from acute injury such as the initial inflammation inciting incident of invasive ocular surgery. Without persistent subsequent immune challenge of xenograft transplantation, as in *non-nude transplant IS* and *non-nude transplant no IS* groups, sham surgery would be essentially self-resolving toward a homeostatic, pre-injury baseline state. To further elucidate this hypothesis, more comprehensive cytokine profiling would be required [21, 24].

Noteworthy is that in both immunosuppressed and immunocompromised groups, we have observed a greater proportion of myeloid and NK cells than in their age and sex matched non-IS treated immunocompetent littermates. We hypothesize that both phenotypes of T-cell deletion and ablation result in similar expansion of the myeloid and NK cell compartment as a physiological compensatory response.

This is substantial, as IFN-γ, a positive feedback instigator of the characteristic ramped up cellular immune response and cytokine storm accompanying acute rejection, is jointly secreted by T-cells *and* NK cells and stimulates phagocytic activity of microglia and macrophages. Therefore, in immune competent IS treated transplant recipients, despite apparent expansion of NK cells and macrophages as result of treatment, upstream T-cell suppression alone proved sufficient in attenuating serum levels of IFN-γ, graft site microglial activation and preserving graft site from infiltration of peripheral macrophages.

Regardless of immune competence status, macrophages and NK cells are inconsequential in precipitating acute rejection of subretinal grafts. This is also exemplified by the fact that not a single case of graft rejection was observed in T-cell deficient athymic nude rats despite such a robust upregulation of myeloid and NK innate immune cells.

In *foxn1−/−* rats, absence of T-cells not only abolishes donor specific cell mediated graft destruction of acute rejection but also T-follicular cells that are responsible for antigen specific education and differentiation of antibody producing B-cells. Though not explicitly measured in our study, this mechanism can extrapolate to the graft tolerance we observed as consequence of successful IS T-cell suppression. T-follicular cells are among the subpopulations of CD4+ T-cells we observed undergoing global proliferative and activation suppression resulting from TAC administration. Thus, both absence and sufficient global attenuation of T-cell populations cripples the antibody mediated humoral immune response which precipitates the longer-term immune phenomenon of chronic graft rejection.

Our results demonstrate that T-cell mediated graft specific cytotoxicity is the primary and essential upstream component of acute rejection of retinal organoid transplants. Cytotoxic lymphocytes destroyed donor cells within weeks, and via positive feedback of IFN-γ and IL-2, drove their own proliferation as well as the chemotaxis and effector functions of activated microglia and peripheral macrophages in response to necrotic donor and host cells. That T-cytotoxic cells are the primary source of graft destruction demonstrates an indirect antigen recognition profile with either one of two probable mechanisms of action:

1) Antigen presenting cells (APCs) in the subretinal space take up antigens (Ag) from graft cells and present to circulating naïve T-cells via endothelial margination through either blood retinal barrier or inner retinal vasculature or 2), shed antigens from donor cells themselves exit the subretinal space initially unchaperoned by APCs to later make their way to draining lymphoid tissues for site specific APC mediated presentation to naïve T-cells. Either scenario results in a T-cell education and subsequent clonal expansion into an effector population with specificity to RO grafts that are then capable of homing in on the original source of their antigen specificity in the subretinal space.

The first APC mediated Ag presentation scenario is noteworthy as it is a sequence that requires cells traversing the often-considered immune privileged subretinal space beyond the blood retinal barrier *to and from* peripheral circulation and lymph nodes at least twice (Supplemental Fig. S4).

Thus, across a continuum of immune-competence states, acute RO graft rejection is only possible with a robust population of functional, sensitized CD8+ and CD4+ effector cells. The typical graft rejection develops as a combination of acute cytotoxic T cell mediated response in case of significant HLA mismatch (including xenogeneic graft) and a delayed antibody mediated rejection in partial or minor HLA matching (i.e., partial matched allogeneic grafts). There is an additional mechanism for acceleration of immune-initiation when the quality of the transplant is low (lot of cell debris). Damage-associated molecular pattern signals are sensed by dendritic cells that will pick-up possible allo-antigens from the debris and present through MHC-II for an antibody response, or by MHC-I cross-presentation to initiate a T-helper 1 (Th1) type response [24]. Lastly, stressed and damaged cells tend to directly express damaged proteins through MHC-I even if HLA is perfectly matched, which can further educate the immune system. Thus, the better the quality of the graft (i.e., viability, lack of mutations, etc.), the more delayed the adaptive response is with better chances of tolerance induction in real-life allogeneic transplants when partial HLA matching is pursued.

Our group and others have observed that potential synaptogenesis of transplanted photoreceptors with bipolar interneurons is associated with grafts that demonstrate functional improvements in visual acuity in optokinetic and electrophysiological assessments in RD animal models. In addition, a subpopulation of Ku80-immunoreactive transplant cells migrated into the degenerative RD host retina.

As the restorative functional capacity of RO transplants is only possible in the context of immune tolerance, suppression of the cytotoxic lymphocyte effector functions is required to achieve successful transplant tolerance in the subretinal space.

### Limitations of this study

Xenograft *non-nude transplant NO IS* response models the worst-case scenario of RO graft rejection. Subsequent studies of this nature may expand the observation time frame of tolerant grafts to obtain a more comprehensive picture of the donor RO’s lifespan with respect to cell proliferation, turnover, and duration of restorative capacity.

Further studies may benefit from a more comprehensive lineage and functionality immune profiling of the upregulated CD161+/CD3− CD8+ lymphocyte and CD68+/CD11bc+ myeloid populations we have observed resulting from T-cell suppression or deletion, respectively. This, in conjunction with expanded microglia, CD4+ T-cell and cytokine profiling would further characterize and elucidate probable mechanisms of the immunoreactivity profiles observed across each tolerance/rejection modality in this study. For example, not explicitly probing for indicators of endogenous immunomodulation such as the presence of CD4+ Tregs and suppressive cytokines (IL10, TGFβ, IFNβ) across surgery modalities (non-surgery, sham, and transplant RD), yields an incomplete picture of the interplay between modulation and inflammation reflective of each corresponding physiological immune environment.

We opted to not pursue LCMS based pharmacokinetic (PK) profiling of MMF. MMF was administered as a secondary supportive immunosuppressant. However, rapid, unequivocal graft rejection in MMF monotherapy transplant cohorts (determined in vivo by OCT imaging 14 days post-transplant) did not warrant symmetrical investment of LCMS/MS mediated serum PK profiling. Furthermore, MMF monotherapy at highest administered dose (300 mg/kg) showed minimal suppression of immune cell populations after 1 and 3 months (data not shown). These results together with the success of 20 mg TAC monotherapy warranted its use at this dose as the empirical efficacy-based anchor for building and evaluation of optimal combination therapy dosing regimens with MMF.

Although TAC and MMF are clinical standard drugs with established safety and efficacy profiles in humans, and they did not show negative effects on our in vitro culture study, they had a lethal impact (cardiac/respiratory arrest) on our in vivo study, resulting in a lower number of rats available for the function tests. The outbreak of Covid 19 pandemic made it worse. During the pandemic, people were restricted from working in the lab, which further reduced the rat number for function tests. Nevertheless, optokinetic testing showed significant improvement in transplanted rats compared to controls at 2–5 m months post-surgery. Especially for SC, only 5 rats with transplants survived to be ready for SC recording but still 3 died in anesthesia before SC recording. Therefore, although the 2 transplant rats did not show responses as has been seen previously with transplants to immunodeficient rats, it is still possible that the other transplants would have elicited a visual response [4].

### Further considerations

While the undisturbed subretinal niche is purported to be an immune protective environment [39], our retinal graft study encounters components essential for its stepwise derangement into an acute rejection phenotype of the highest order; a primed pro-inflammatory environment of retinal degeneration exacerbated by the transplantation induced tissue trauma, (and possible blood retinal barrier breach) of an invasive surgery delivering a therapeutic containing a sufficiently immunogenic foreign antigen.

The predictive efficacy of in vitro alloreactivity of CSC14 cells is informative, but incomplete without an in vivo component. Factoring out the in vivo component of allo-immunoreactivity minimizes the systemic cumulative physiological interactions that grant an authentically embodied adaptive immune response all its potency. For example, in vitro, weak HLA I expression on hESC ROs does not guarantee immunity to allorecognition. In principle, any non-self-generated cellular antigen in vivo can elicit at least foreign-body, MHC-II based adaptive cell mediated and humoral responses, respectively, in immunocompetent individuals.

An essential progression of these studies will be in evaluating RO allograft immunoreactivity in vivo, with a competent “humanized immune system” animal model. The humanized immune system animal model is regarded as an aspirational, functional gold standard of sorts for assessment of pre-clinical translational immunoreactivity of potential stem cell-based therapies. This said, in the context of personalized medicine, in vitro methods like HLA matching and MLR assay may yield the highest predictive value and content information in terms of direct immunoreactivity profiling of individual prospective graft recipients. We used human PBMCs in the MLR assay to mimic the allograft situation in a future patient. Such an approach gains increased relevance with optimization of iPSC-tissue derivation methods.

The successful precedent of TAC and MMF treatment in promoting immune tolerance in solid organ transplants made an adequate choice for preliminary investigation of xenograft tolerance. Still, our drugs of choice in this study may not be the most appropriate for stem cell derived photoreceptor transplantation to the subretinal space in human subjects. Sevc et al. and others have done foundational work in exploring immune suppression in preclinical stem cell replacement-based xenograft studies in immune competent disease models [30]. Ultimately, we acknowledge the lack of immunogenic parity between xenografts and allografts in our experiments.

Graft tolerance therapeutic strategies based on administration of TAC and MMF were initially developed in the context of the immunogenic profiles of solid organ transplants. Such transplants have the antigenic richness of solid organs composed of multiple tissue functional units, many embedded with tissue resident immune cells, and developed within individuals with distinct HLA profiles. ROs as isolated tissue systems derived from off-the-shelf pluripotent stem cell lines in vitro inherently lack the antigenic richness of solid organs. Additionally, medical conditions behind the nature of such transplants, heart, kidney, lung, or liver, respectively, carry sufficient threat of severe impairment or of loss of life as to warrant incurred risks of drug-induced organ toxicity and/or opportunistic infection. Retinal degeneration, while detrimental to quality of life, is not a comparable class of life-or-death disease, and as such, warrants less drug-induced risk from the same drug regimen.

The toxicity profile and vulnerability delivered by immunosuppressants must not yield more potential risk than the efficacy profile of any proposed therapeutic intervention or treatment. Other, lower risk options for attenuating cell mediated response (checkpoint inhibition, glucocorticoids, administration of exogenous immunosuppressive cytokines, anti-thymocyte globulin) may be more adequate and are worth exploring.

The uniquely attenuated immunoreactivity profile of stem cell derived tissue and the subretinal niche, rise in optimization of iPSC derivation, and burgeoning shift in regenerative medicine and immunology research focusing on endogenous, engineered, and induced immunomodulation in advance of traditional chemical immunosuppression, render this type of treatment particularly amenable to actionable and testable refinements in domains of both safety and efficacy.

## Conclusion

Restorative capacity of this and any tissue replacement therapy is conditionally dependent upon host tolerance. Any measures taken to promote graft tolerance must do no net harm to the prospective host. As such, preliminary investigations of this nature at the intersection of tolerance, safety and functional efficacy are necessary.

In the context of an immunoreactive environment, T-cell mediated immune recognition is necessary for acute rejection, and our study determines the suppression of this response as proximally essential for graft survival and thereby full access to the restorative potential of photoreceptor cell replacement therapy.

The ongoing challenge of this and further studies of this nature will be in achieving optimal restorative capacity while minimizing the cost of tolerance, i.e., all the concomitant vulnerabilities of graft recipient becoming chemically immune compromised and subject to toxic side effects of immunosuppressive drugs.

## Supplementary Information

Additional file 1

## Data Availability

The datasets used and/or analyzed during the current study are available from the corresponding author on reasonable request.

## Abbreviations

Ag: Antigen
AMC: Age-matched control (no surgery)
APC: Antigen presenting cell
CTL: Cytotoxic lymphocytes
DPS: Days post-surgery
hESC: Human embryonic stem cell
HLA: Human leukocyte antigen
IFN-γ: Interferon gamma
IL-2: Interleukin 2
IS: Immunosuppression
MHC: Major histocompatibility antigen
NK cell: Natural Killer cell
MMF: Mycophenolate Mofetil
MPA: Mycophenolic acid
NN: Non-nude immunocompetent RD rat
NN AMC: Immunocompetent age matched control RD rat
Nude: Immunocompromised RD rat (no T-cells)
PBMCs: Peripheral blood mononucleate cells
PK: Pharmacokinetic
PR: Photoreceptor
RD: Retinal degenerate (or retinal degeneration)
RO: Retinal organoid
RP: Retinitis Pigmentosa
Sham: Immune competent RD rat; subretinal injection of RO culture media only
TAC: Tacrolimus
TP: Transplant. Denotes any RO graft recipient
TP IS: NN RD rat receiving RO graft and immunosuppressive treatment
TP NO IS: NN RD rat receiving RO graft and no immunosuppressive treatment
Treg: Regulatory T-lymphocyte
WT: Wildtype

## Code availability

Matlab code for analysis of superior colliculus responses is available upon request.
