# Supplementary material for: Effect of immunosuppression on hESC-derived retina organoids in vitro and in vivo
Source: Stem Cell Res Ther. 2025 Apr 5;16:165. doi: 10.1186/s13287-025-04271-z (PMC11972460; doi:10.1186/s13287-025-04271-z)
Supplement: Supplementary file 1 — Additional file 1 [file 13287_2025_4271_MOESM1_ESM.docx]

**Supplemental Materials: Effect of immunosuppression on hESC-derived retina organoids *in vitro* and *in vivo***

Sims, Robert^1,2,3,4,5^†; Lin, Bin^1, 2,3,4,5^†; Xue, Yuntian ^1,6^, Fouda, Raghda^1^, McLelland, Bryce T.^3^; Nistor, Gabriel^3^; Keirstead, Hans S.^3^; Browne, Andrew W. ^2,5,6,7^, Seiler, Magdalene J. ^1,2,3,4,5^

The following supporting information contains additional methods, results and tables in support of the main text. This document contains 3 figures and 4 tables.

**Supplemental Methods:**

***Hematoxylin-Eosin Staining:***

Slides with frozen sections were placed in Bouin’s fixative (Polysciences, Cat. #16045) for at least 1 hour or overnight, washed 2 times with 70% ethanol (10 dips each), then rinsed 2 times with deionized water before being placed into filtered Hematoxylin (Harris Hematoxylin, Sigma HHS-32) for 12 min., followed by a rinse with tap water, 2 quick dips in acid ethanol (20 ml 50% HCl in 1 L 70% ethanol) (for differential nuclear staining). Then slides were rinsed with tap water, then placed in Ammonia water (3 ml NH_3_ in 1L tap water) for at least 3 min., followed by rinsing with double-distilled water, and 10 dips 70% ethanol. Then, slides were placed into Eosin (Eosin Y solution, alcoholic, Cat. # HT110-32, Sigma; or Cat. # ab246824, Abcam) for maximal 40 seconds, dehydrated (20 dips each) in 95% Ethanol I and II, 100% Ethanol (I, II, III), followed by 100% Clear-Rite I and II (20 dips each) and then placed into Clear-Rite III for at least 15 min. (up to overnight) for coverslipping with 50% Permount in Clear-Rite (all chemicals from Fisher Scientific unless noted otherwise).

This procedure resulted in blue staining of nuclear layers (photoreceptor layer darker than other layers) and pink staining of plexiform layers,


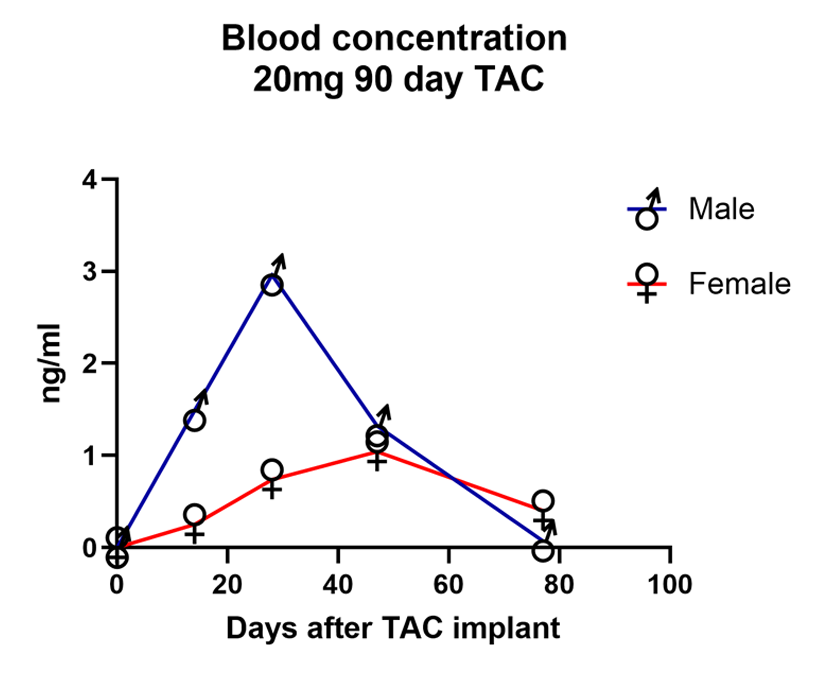


**Supplemental Figure S1. Therapeutic drug monitoring of tacrolimus 20mg 90-day release absorption and elimination profiles – individual data.**

Individual tacrolimus PK profiles of each (2 male, 2 females) dosing group of immunocompetent RD rats across treatment period. Note individual variability.


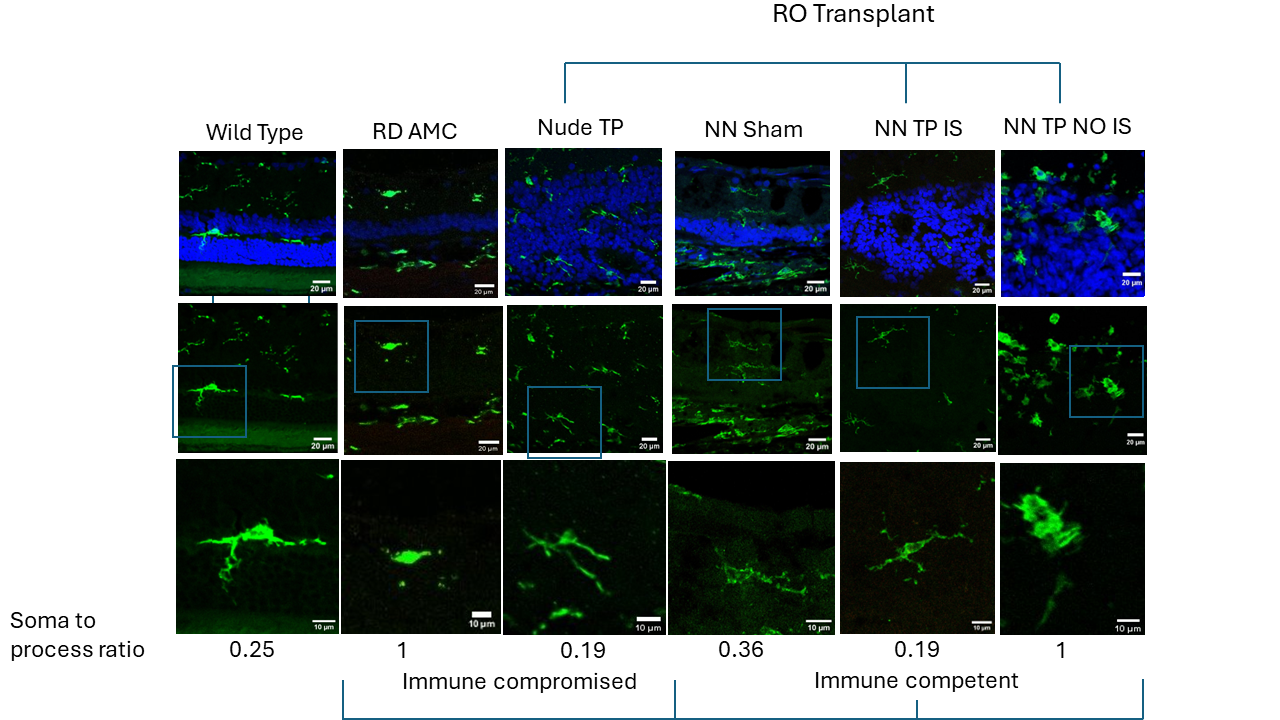


**Supplemental Figure S2:** **Overview of observed microglia morphologies across disease, surgery, and treatment modalities:**

From left to right: Iba1+ Microglia in wild type retina, nude RD (untreated), nude RD transplant, immune competent receiving sham surgery, IS treated immune competent transplant, and non-IS treated immunocompetent transplant.

Where branching processes and cell soma size are qualitative morphological indicators of microglia activation status, soma to process length ratios approaching 1 were more associated with microglia in inflammatory, reactive and phagocytic states. Here, microglia examples from late-stage RD in immune compromised (Nude TP) and non-IS treated (NN TP IS) immune competent transplant recipient experiencing acute graft rejection alike, display retracted processes and enlarged soma, whereas examples from immune compromised and immune suppressed graft recipients demonstrate similar microglia soma to process ratios.


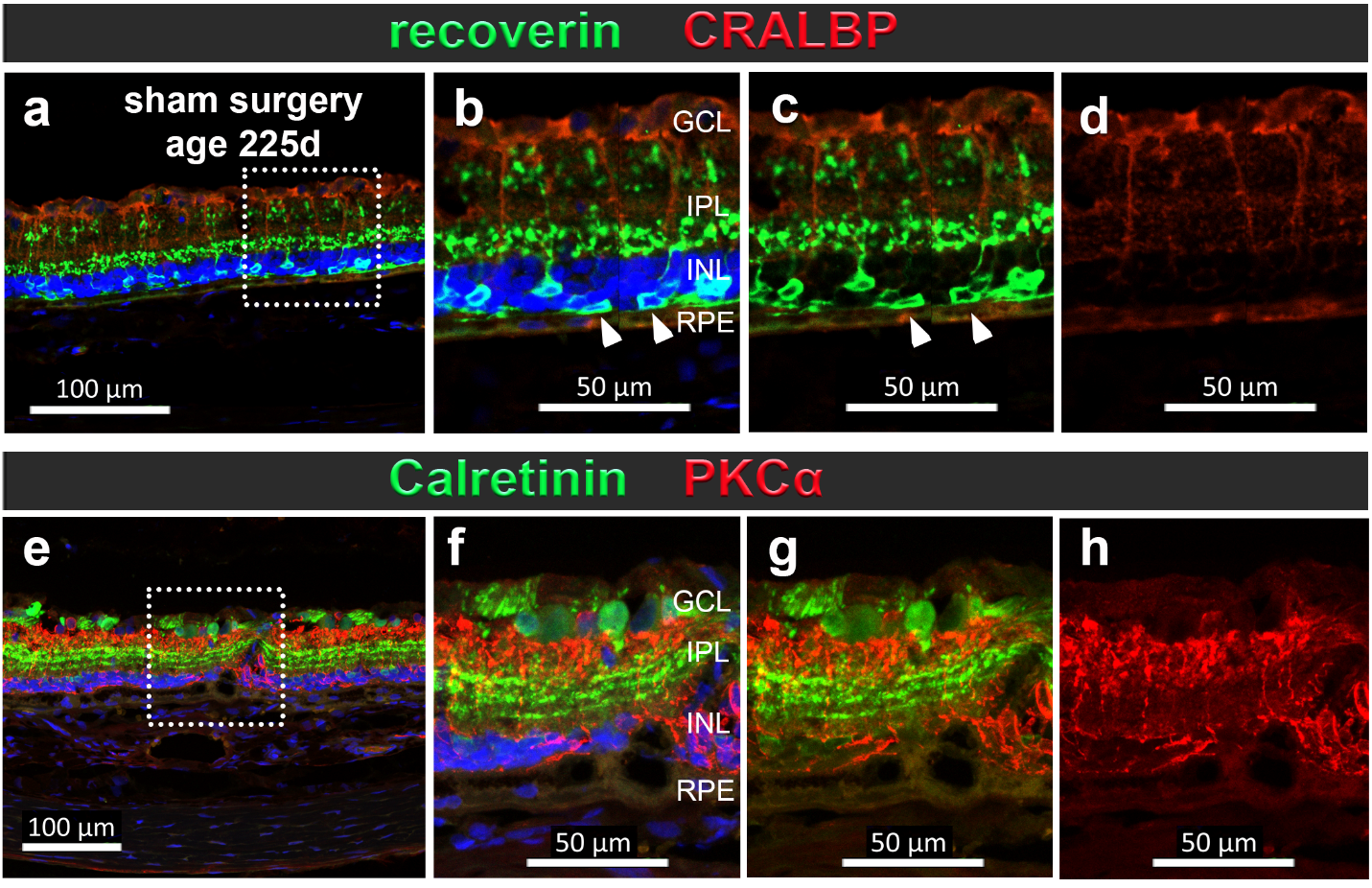
**Supplemental Figure S3: Retinal markers in RD rat with advanced retinal degeneration.**

RhoS334ter-3 rat, age 225d, 188 days post-surgery. This rat was recorded in the SC with no response. Nuclei are labeled blue (DAPI). **a)-d)** recoverin (green): Few remaining cones with abnormal morphology left (arrowheads). Cellular retinaldehyde binding protein (CRALBP, red): retinal Müller cells.  **e)-h)** Calretinin (green): retinal ganglion and some amacrine cells. Protein kinase C α (PKC α, red): rod bipolar cells. Not the remodeling and distortion in the INL (blood vessel).


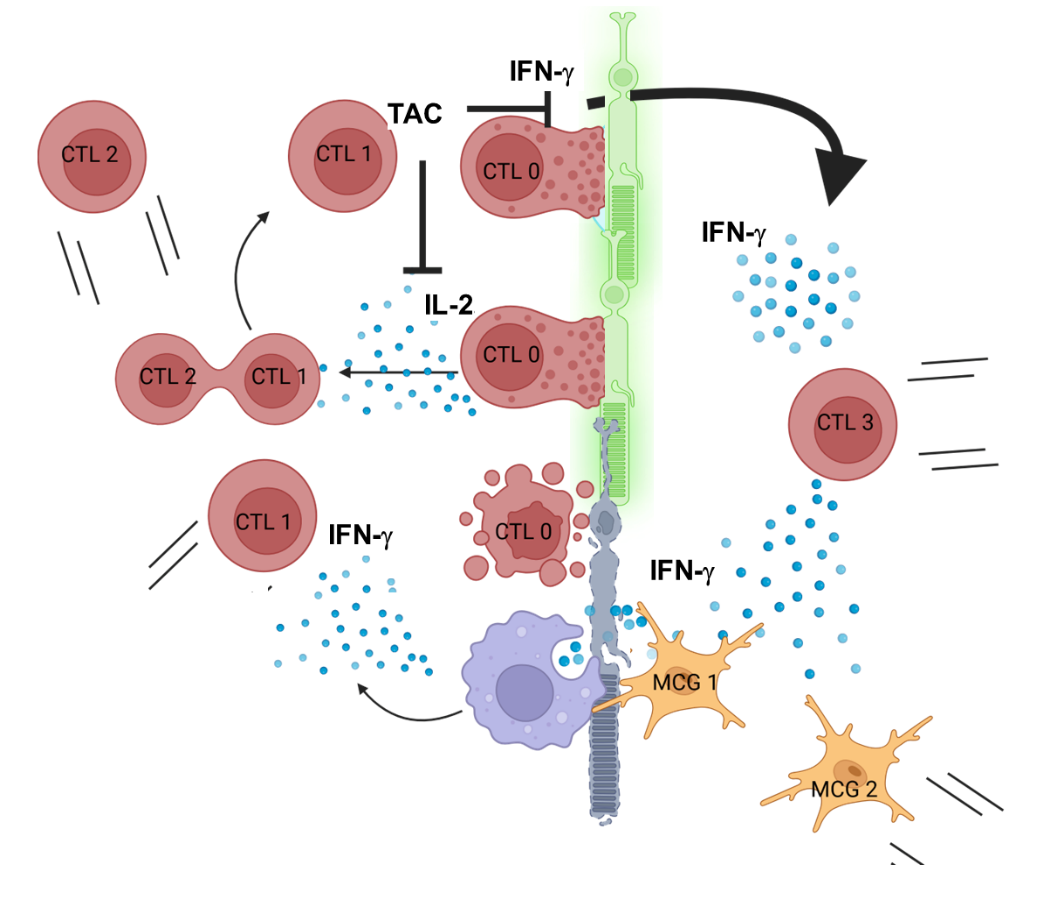


**Supplemental Figure S4: Immune Cell Infiltration and graft cell rejection:**

Graft rejection occurs in non-immunosuppressed, immune competent graft recipients (NN TP IS). Activated, RO antigen specific CD8+ CTLs infiltrate the subretinal space and kill grafted photoreceptors. CTL secrete IFN-γ and IL-2 further their own proliferation. IFN-γ additionally activates microglia and macrophages involved in the phagocytosis of apoptotic graft cells and exhausted CTLs alike.

This sequence outcomes characteristic of Xenograft rejection is averted upstream by sufficient tacrolimus mediated suppression of the activation and proliferation of RO antigen sensitized CTLs.

**Supplemental Table S1: List of Components Used in the Retinal Organoid Manufacturing Process**

| **Reagent** | **Purpose** | **Composition** | **Concentration at use** | **Quality COA** | **Grade** | **Source** | **Vendor** |
| --- | --- | --- | --- | --- | --- | --- | --- |
| ABStem Supplement | Supplement in the media composition for stem cell expansion | Chemically defined | 10% v/v | Yes | GMP | Xeno-free | FUJIFILM Irvine Scientific |
| ABStem basal media | Media for stem cell growth | Chemically defined | 90% | Yes | GMP | Animal-free | FUJIFILM Irvine Scientific |
| Collagenase IV solution | Enzyme for stem cell dissociation | Collagenase IV | 0.002 | Yes | Cell culture | Bacteria | Thermo Fisher Scientific |
| Growth factor supplement | Growth factors for pluripotent stem cell expansion | Basic FGF | 10 ng /mL | Yes | GMP | Recomb | Peprotech |
|  |  | Activin-A | 5 ng/mL | Yes | Cell culture | Recomb | Peprotech |
| Matrigel | Substrate for stem cell culture | Matrigel | 0.01% | Yes | Cell culture | Mouse | Thermo Fisher Scientific |
| Freezing media | Media used to freeze stem cells | Dimethyl Sulfoxide (DMSO) | 10% | Yes | USP | Synth. | Sigma |
|  |  | ABStem | 90% | Yes | GMP | Animal-Free | FUJIFILM Irvine Scientific |

**Supplemental Table S2: Antibodies used in this study**

| **S2.A. Primary antibodies (IHC)** | | | | | | |
| --- | --- | --- | --- | --- | --- | --- |
| **Antigen** | **species** | **specific for** | **dilution** | **Supplier** | **Catalogue #** | **RRID** |
| Calretinin (SP13) | rabbit | Ganglion and amacrine cells | 1:200 | Thermofisher | MA5-14540 | AB_10985167 |
| Calretinin | goat | Ganglion and amacrine cells | 1:100 | Novus Biologicals (previously R&D systems) | AF5065 | AB_2824405 |
| Caspase 3 (cleaved) | rabbit | Intercellular Caspase 3 | 1:200 | StressMarq | SPC-1319D | AB_2737391 |
| CRALBP | mouse | Muller Glia, RPE | 1:200 | Abcam | Ab15051 | AB_2269474 |
| CD4 (W3/25) | mouse | T-helper cells | 1:200 | BioRad | MCA55 | AB_2075686 |
| CD8α | mouse | Cytotoxic t-cells | 1:200 | BioRad | MCA609 | AB_321407 |
| CD45 (YW62.3) | mouse | Pan Lymphocyte | 1:200 | BioRad | MCA1031 | AB_321730 |
| CD68 (ED1) | mouse | Macrophages, Monocytes | 1:200 | BioRad | MCA341 | AB_2291300 |
| CD161 (10/78) | mouse | Natural killer lymphocytes | 1:200 | BioRad | MCA1427 | AB_2234351 |
| Human specific synaptophysin | mouse | human neuronal processes & synapses | 1:400 | ThermoFisher | 14-6525-82  (EP-10) | AB_2574270 |
| Iba-1 | rabbit | Microglia | 1:100 -1:200 | Biocare Medical (Pacheco, CA) | CP 290 A | AB_10578940 |
| KI67 (RM360) | rabbit | Nuclear mitotic spindle | 1:200 | BioRad | MCA6357 | P46013 |
| Ku80 | rabbit | Human nuclei | 1:400 | Abcam (Eugene, OR) | ab80592 | AB_1603758 |
| MHC II RT1B (OX6) | mouse | MHC II receptor | 1:200 | Biorad | MCA46GA | AB_567369 |
| PKCα (H-7) | mouse | Rod bipolar cells | 1:200 | Santa Cruz | SC-8393 | AB_628142 |
| PKCα (MC5) | mouse | Rod bipolar cells | 1:200 | Invitrogen | MA-157 | AB_2536865 |
| RAX | rabbit | Retinal progenitor cells | 1:500 | Abcam | Ab86210 | AB_1925380 |
| Recoverin | rabbit | Photoreceptors, cone bipolar cells | 1:2K (fl. Ab)  1:10K (ABC) | Millipore | AB5585 | AB_2253622 |
| Rhodopsin (rho4D2) | mouse | Rods | 1:100 (fl. Ab)  1:10K (ABC) | Dr. Robert Molday, Univ. of British Columbia (40) | N/A | N/A |

| **S2.B. Secondary Antibodies** | | | | | | |
| --- | --- | --- | --- | --- | --- | --- |
| **Conjugate** | **species** | **specific for** | **dilution** | **supplier** | **Catalogue #** | **RRID** |
| Alexa Fluor 488 | Donkey | Rabbit IgG (H+L) | 1:400 | Jackson Immuno Research (West Grove, PA) | 711-545-152 | AB_2313584 |
| Rhodamine Red-X | Donkey | Mouse IgG (H+L) | 1:400 | Jackson Immuno Research | 715-295-151 | AB_2340832 |

| **S2.C. Flow Cytometry** | | | | | | | |
| --- | --- | --- | --- | --- | --- | --- | --- |
| **Conjugate** | **species** | **specific for** | **dilution** | **supplier** | **Catalogue #** | **RRID** |  |
| FITC | rabbit | OTX2 | 1:100 | Millipore-Sigma | AB9566 | AB_2157186 |  |
| FITC | mouse | Ki67 | 1:100 | eBioscience | 11-5699 | Not available* |  |
| Pacific Orange | mouse | Human CD45 | 1:100 | Biolegend | Not available* | Not available* |  |
| PE/Cy5 | mouse | Human CD3 | 1:100 | Biolegend | 300409 | AB_314063 |  |
| AF488 | mouse | Human CD4 | 1:100 | Biolegend | 344617 | AB_10559751 |  |
| PE-Cy7 | mouse | Human CD8 | 1:100 | Biolegend | Not available* | Not available* |  |
| FITC | mouse | Human MHC-I | 1:100 | Biolegend | Not available* | Not available* |  |
| APC/Cy7 | mouse | Human MHC-II | 1:100 | Biolegend | Not available* | Not available* |  |
| APC | mouse | Rat CD45RA (OX-33) B-cells | 1:100 | Biolegend | 202314 | AB_10639733 |  |
| APC | mouse | Rat CD161 NK cells | 1:100 | Biolegend | 205606 | AB_11142680 |  |
| BV 421 | N/A | Viability Dye | 1:100 | Biolegend | 423113 | Not available |  |
| FITC | mouse | Rat CD3+ Pan T-cells | 1:100 | Biolegend | 201403 | AB_2073344 |  |
| PE | mouse | Rat CD8a (OX-8) Cytotoxic T-cells | 1:100 | Biolegend | 201705 | AB_2075262 |  |
| PE | mouse | Rat CD11b/c (OX-42) Granulocytes/Macrophages | 1:100 | Biolegend | 201807 | AB_313994 |  |
| PE-Cy7 | mouse | Rat CD4 (W3/25) T-helper cells | 1:100 | Biolegend | 201516 | AB_1186089 |  |

* Product discontinued; could not be found on supplier and/or RRID website

**Supplemental Table S3: Information of qPCR primers
(Qiagen Quantitect primers)**

| Gene name | Official full name | GeneGlobe ID |
| --- | --- | --- |
| OCT4 | Octamer-binding transcription factor 4 (self-renewal of hESCs) | QT00210840 |
| CHX10 (VSX2) | Visual system homeobox 2 | QT00221081 |
| CRX | Cone-rod homeobox | QT01192632 |
| NRL | Neural retina leucine zipper | QT01005165 |
| RAX | Retina and anterior neural fold homeobox | QT00212667 |
| RCVRN | Recoverin | QT00014098 |
| ARR3 | Arrestin 3 | QT00000182 |
| GNAT | G-protein subunit alpha transducin | QT00235606 |
| GNAT2 | G-protein subunit alpha transducin 2 | QT00008764 |
| SAG | S-antigen visual arrestin | QT01007958 |
| RHO | Rhodopsin | QT01017058 |
| OPN1SW | Opsin 1, short wave sensitive | QT00017304 |
| OPN1MW | Opsin 1, medium wave sensitive | QT00040887 |
| OPN1LW | Opsin 1, long wave sensitive | QT01007356 |
| PRPH2 | Peripherin 2 | QT00094094 |
| ACTB | Actin beta (human) (housekeeping gene) | QT00095431 |
| RPL7 | Ribosomal protein L7 (housekeeping gene) | QT01670137 |

**Supplemental Table S4: statistical comparison of drug-treated organoids with controls**

| **Sample** | **Comparison** | **P-value** | **Sig. Difference** |
| --- | --- | --- | --- |
| TR 52 1 wk | Ctrl vs. MPA | >0.9999 | No |
|  | Ctrl vs. TAC | 0.6653 | No |
|  | Ctrl vs MPA + TAC | 0.4048 | No |
| TR 52 4 wks | Ctrl vs. MPA | 0.8373 | No |
|  | Ctrl vs. TAC | 0.7071 | No |
|  | Ctrl vs MPA + TAC | 0.9557 | No |
| TR 61 1 wk | Ctrl vs. MPA | 0.3265 | No |
|  | Ctrl vs. TAC | 0.9983 | No |
|  | Ctrl vs MPA + TAC | 0.9999 | No |
| TR 61 4 wks | Ctrl vs. MPA | 0.9896 | No |
|  | Ctrl vs. TAC | 0.9937 | No |
|  | Ctrl vs MPA + TAC | 0.9982 | No |
